# Fruit and vegetable consumption frequency and tooth-loss transitions across baseline dentition strata in older Chinese adults: a prospective cohort study

**DOI:** 10.3389/fnut.2026.1829512

**Published:** 2026-07-17

**Authors:** Kan Xu, Xiaojuan Su, Wenqiang Lu, Ziqi Yang, Yihao Pei, Changjiang Wu, Yiming Mao, Fang Li, Ying Yuan

**Affiliations:** 1 Department of Geriatrics, Zhongshan Hospital, Fudan University, Shanghai, China; 2 Department of Geriatrics, Zhongshan Hospital (Xiamen), Fudan University, Xiamen, China; 3 Department of Thoracic Surgery, Suzhou Kowloon Hospital, Shanghai Jiao Tong University School of Medicine, Suzhou, China; 4 School of Stomatology, Medical College of Jinzhou Medical University, Jinzhou, China; 5 Department of Intensive Care Unit, Suzhou Kowloon Hospital, Shanghai Jiao Tong University School of Medicine, Suzhou, China

**Keywords:** Chinese Longitudinal Healthy Longevity Survey, cohort study, edentulism, fruit consumption frequency, older adults, Oral health, tooth loss, vegetable consumption frequency

## Abstract

**Background:**

Fruit and vegetable consumption is linked to oral health in later life, but whether consumption frequency is associated with subsequent tooth-loss transitions across baseline dentition stages remains unclear. We examined these associations in older Chinese adults.

**Methods:**

We used Chinese Longitudinal Healthy Longevity Survey data from 2008, 2011, 2014, and 2018. Participants aged ≥65 years were grouped by baseline dentition; the outcome was incident edentulism in the 1–19-teeth stratum and transition to fewer than 20 teeth in the 20–24 and 25–32-teeth strata. Models mutually adjusted fruit and vegetable consumption frequency. Target-population interpretation prioritised a full baseline-eligible cohort competing-outcome analysis treating death as a competing outcome; additional analyses addressed alternative exposure definitions, differential inclusion, and competing survival.

**Results:**

In the full baseline-eligible cohort competing-outcome analysis among participants with 1–19 baseline teeth, more frequent vegetable consumption showed an attenuated inverse association with incident edentulism after accounting for death as a competing outcome (HR 0.88, 95% CI 0.73–1.04). In the observed-outcome sample with follow-up tooth counts, the corresponding vegetable estimate was stronger (HR 0.83, 95% CI 0.69–0.99), whereas fruit consumption was not associated with incident edentulism (HR 0.97, 95% CI 0.86–1.09). Associations in the 20–24 and 25–32 baseline-teeth strata were weaker and less consistent.

**Conclusion:**

Among older Chinese adults with 1–19 baseline natural teeth, higher vegetable consumption frequency showed only a weak inverse association with incident edentulism when the full baseline-eligible cohort and competing death were considered, whereas fruit consumption remained near null. The stronger estimate from the observed-outcome sample may overstate the association in the target population and should not be interpreted as evidence of an independent protective effect.

## 1 Introduction

Tooth loss and edentulism remain major challenges in ageing populations because they are common and can substantially affect daily function and quality of life. The World Health Organization has framed oral health as an integral part of healthy ageing rather than a domain separate from general health (1). Recent global estimates further underline the scale of this burden, showing that oral conditions continue to impose a substantial burden worldwide and that edentulism remains an important source of disability in later life (2). In geriatric dentistry, retention of 20 or more natural teeth remains a widely used benchmark for functional dentition (3). This benchmark is clinically relevant because tooth retention has been linked to better health and quality of life in older adults, and the presence of at least 20 natural teeth has also been associated with more favourable survival outcomes (4, 5). Longitudinal evidence increasingly places oral health within the broader framework of healthy ageing, reinforcing the need to study tooth-loss trajectories as part of later-life health rather than as an isolated dental outcome (6).

Diet may be relevant to these trajectories, although the available evidence remains largely associational and the relationship with oral health is likely bidirectional in older adults. Reviews in older adults consistently show that oral health, diet, and nutritional status are closely interconnected (7). Older adults with fewer teeth tend to consume fruits and vegetables less frequently (8), and in Chinese older adults, a lower number of natural teeth has likewise been associated with poorer dietary diversity and less favourable nutritional status (9). Recent review-level evidence also suggests that healthier dietary patterns are associated with more favourable periodontal status (10). Because periodontal breakdown is a well-established driver of later tooth loss, and more advanced periodontitis has been associated with substantially higher rates of subsequent tooth loss during maintenance care (11), fruit and vegetable consumption frequency may therefore be relevant to subsequent tooth-loss trajectories, while also remaining susceptible to reverse causation and broader confounding by oral, functional, and social vulnerability.

That question, however, remains insufficiently resolved in older populations, particularly when baseline dentition is already reduced. A longitudinal study in Chinese older adults reported that dietary habits were associated with the subsequent number of natural teeth (12). That was an important step forward, but modelling tooth count alone may obscure clinically distinct transitions in oral status. A decline from 22 teeth to 19 and progression from 8 teeth to none are not equivalent events. They represent different stages of oral deterioration, with different functional implications and different degrees of clinical irreversibility. Interpretation becomes still more complicated once dentition is already reduced, because prosthetic rehabilitation may partly compensate for loss of oral function without restoring natural dentition or fully restoring eating patterns (13). A more clinically informative approach is therefore to examine tooth-loss transitions within predefined baseline dentition strata rather than treating all tooth loss as a single continuum.

This question is important in older Chinese adults because tooth-loss progression occurs within a setting shaped by reduced dentition, unequal dental service use, denture rehabilitation, and dietary behaviours influenced by oral function and socioeconomic conditions. Whether fruit and vegetable consumption frequency is associated with later tooth-loss transitions in this setting remains unclear, particularly among adults whose dentition is already reduced. We therefore examined the association between baseline fruit and vegetable consumption frequency and subsequent tooth-loss transitions in older Chinese adults. The CLHLS provided a nationwide longitudinal cohort with repeated tooth-count and dietary-frequency data suitable for addressing this question.

## 2 Materials and methods

### 2.1 Data source and study design

We conducted a prospective cohort study using data from the Chinese Longitudinal Healthy Longevity Survey, a nationwide longitudinal study of older adults in China (14). We treated the 2008 wave as baseline and followed participants through the 2011, 2014, and 2018 waves. The analysis was organised according to predefined baseline dentition strata rather than a single pooled cohort because the clinical meaning of subsequent tooth loss differs substantially according to baseline tooth count.

### 2.2 Study population and predefined baseline dentition strata

Participants first had to be aged 65 years or older at baseline and to have baseline information on natural teeth count available. We then classified participants into three predefined baseline dentition strata. The primary analysis included participants with 1–19 baseline teeth and evaluated progression to incident edentulism. The two secondary analyses included participants with 20–24 baseline teeth and 25–32 baseline teeth, respectively, and evaluated transition to fewer than 20 teeth. Because the endpoints differed across strata, estimates were interpreted as stage-specific transition estimates rather than directly comparable hazard ratios across cohorts.

The primary analysis focused on participants with 1–19 baseline teeth because progression from partial dentition to complete tooth loss represents the most clinically discrete transition among the three baseline states. The two secondary analyses were retained to assess whether similar associations were present at earlier stages of tooth-loss progression. Participants with 0 baseline teeth were not eligible because no further tooth-loss transition could be defined.

### 2.3 Exposure assessment

Self-reported fruit and vegetable consumption frequency was assessed using standard CLHLS questions. For the primary analysis, fruit consumption frequency was classified as higher when participants reported eating fruit almost every day or quite often and as lower when they reported eating fruit occasionally or rarely or never. Vegetable consumption frequency was classified as higher for the two most frequent response categories, “almost every day” and “almost every day except in winter,” and as lower when participants reported eating vegetables occasionally or rarely or never. This binary grouping followed the CLHLS response structure; in particular, the “almost every day except in winter” category may not represent the same year-round intake pattern as “almost every day.” The principal models included fruit consumption frequency and vegetable consumption frequency simultaneously, allowing estimation of their mutually adjusted associations. Baseline exposure was retained as the primary specification because the main question concerned whether dietary frequency reported at cohort entry was associated with subsequent tooth-loss transitions. In older adults with declining dentition, updating diet during follow-up may partly capture downstream changes related to tooth-loss progression, chewing difficulty, frailty, or preterminal decline. Time-updated and cumulative-average exposure definitions were therefore treated as sensitivity analyses rather than primary specifications. Accordingly, stronger estimates in time-updated or cumulative-average analyses were interpreted as compatible with changes in diet following oral or functional decline rather than as dose-response evidence. To assess whether binary coding discarded important information from the original response categories, additional analyses in the primary cohort used the original ordered fruit and vegetable frequency categories in ordinal trend and four-level factor models. Joint four-category exposure models were retained as exploratory analyses.

### 2.4 Outcomes and follow-up

Self-reported natural teeth counts were available at each wave. In the primary analysis, the outcome was incident edentulism, defined as 0 natural teeth at a follow-up wave among participants who had 1–19 baseline teeth. In the two secondary analyses, the outcome was incident loss of functional dentition, defined as fewer than 20 natural teeth at a follow-up wave. Follow-up was divided into three intervals: 2008–2011, 2011–2014, and 2014–2018. For each analysis, participants contributed interval-specific observations only when end-of-interval tooth-count data were observed. If tooth-count data were missing at the end of a given interval, participants were censored at the start of that interval and did not contribute subsequent intervals. After the first event, no further intervals were included.

### 2.5 Covariates

Baseline covariates were selected a priori and included age, sex, education, marital status, residence, current smoking, current alcohol drinking, regular exercise, sleep duration, body mass index category, activities of daily living limitations, denture use, hypertension, diabetes, heart disease, and stroke. Baseline natural teeth count was additionally adjusted for within each baseline dentition stratum. Categorical covariates were represented with explicit missing categories during model fitting, whereas missing categories were not shown as separate rows in the descriptive tables for readability.

### 2.6 Statistical analysis

We summarised baseline characteristics by baseline vegetable consumption frequency in the primary cohort and in the two secondary cohorts. We then created person-period datasets and fitted discrete-time complementary log-log models with interval indicators and an offset for log interval length. The minimal model adjusted for age and sex. The primary model additionally adjusted for education, marital status, residence, smoking, drinking, exercise, sleep duration, activities of daily living limitations, body mass index category, baseline tooth count, and denture use. The extended model further adjusted for hypertension, diabetes, heart disease, and stroke. Robust standard errors were clustered by participant. Within the 1–19 baseline-teeth stratum, selection and competing survival were addressed through three complementary approaches: a full baseline-eligible cohort competing-outcome analysis, inclusion weighting for entry into the observed-outcome sample, and death-related composite and death-only endpoints. The full baseline-eligible analysis was prioritised for target-population interpretation; it retained participants regardless of later tooth-count observation, handled death as a competing outcome, and was repeated with additional adjustment for self-rated local economic status (f34). The observed-outcome analysis was retained to describe incident edentulism among participants with observed follow-up tooth-count data. Additional sensitivity and exploratory analyses examined alternative exposure definitions, additional adjustment for Parkinson’s disease and epilepsy, joint exposure models, denture-stratified models, complete-case analysis, original ordered exposure categories, interval-specific follow-up status, and interval-stability and model-fit diagnostics. All analyses were conducted in R version 4.5.1. The analytic code used in this study is available from the corresponding authors upon reasonable request.

## 3 Results

### 3.1 Participants

**Figure 1:**
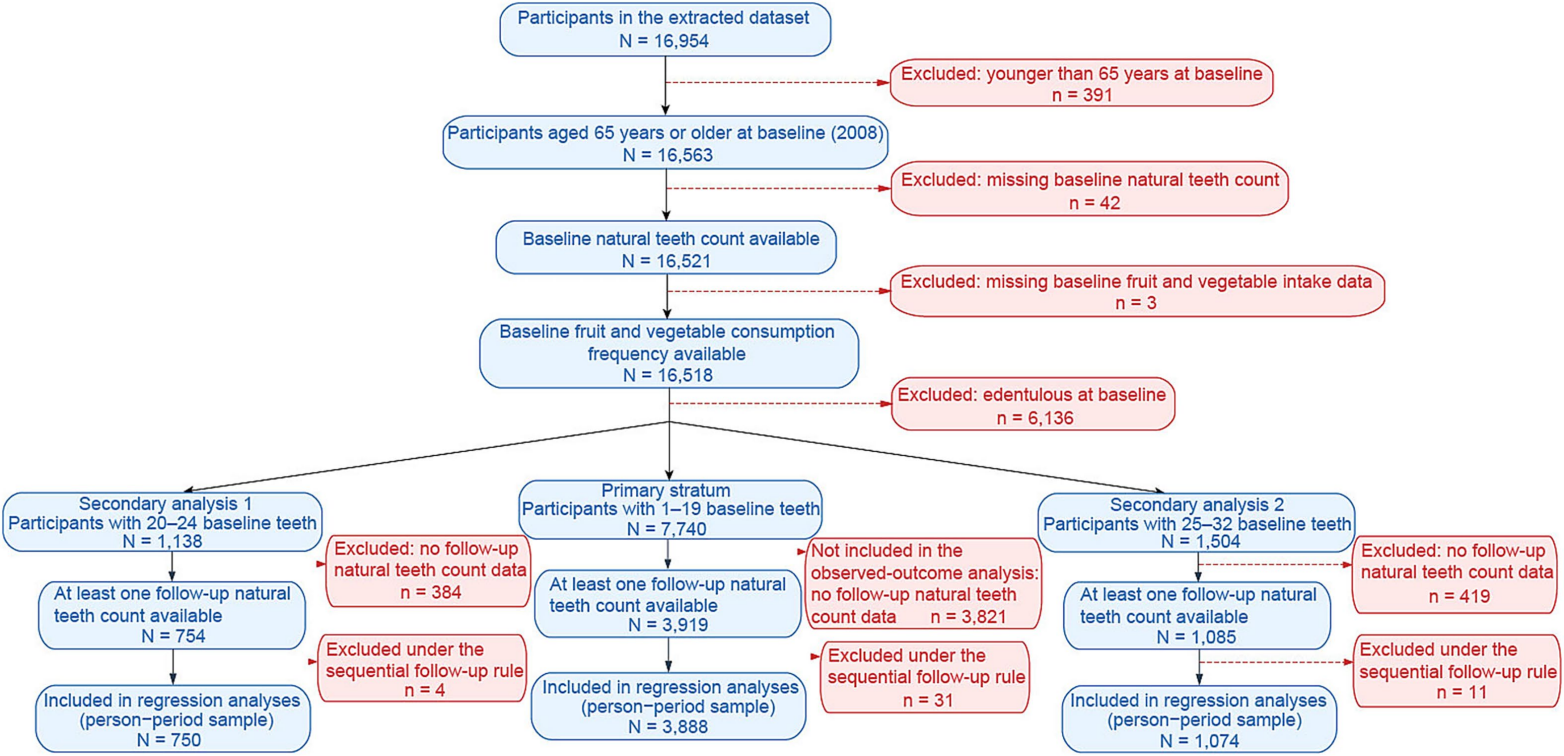
Flow diagram of participant selection and construction of the primary and secondary analysis cohorts defined by baseline dentition status. In the 1–19-teeth stratum, the full baseline-eligible cohort (*n* = 7,740) was used for the competing-outcome analysis; the lower branch depicts selection into the observed-outcome person-period analysis.

Figure 1 summarises participant selection and construction of the three predefined baseline dentition strata. After restriction to participants aged 65 years or older with baseline natural teeth data and baseline fruit and vegetable consumption frequency data, and after exclusion of those who were edentulous at baseline, the three analysis cohorts were defined. Among these, 754 participants in the secondary analysis with 20–24 baseline teeth, 3,919 in the primary analysis with 1–19 baseline teeth, and 1,085 in the secondary analysis with 25–32 baseline teeth had at least one follow-up natural teeth count available. After application of the sequential follow-up rule in the person-period datasets, 750, 3,888, and 1,074 participants, respectively, were included in the regression analyses. In the primary 1–19-teeth stratum, participants excluded before the observed-outcome analysis were substantially older than included participants (median age 92 vs. 83 years) and had less favourable baseline profiles, including less regular exercise, more ADL limitation, lower baseline tooth counts, and lower denture use (Supplementary Table S2).

### 3.2 Baseline characteristics of the primary cohort

**Figure 2:**
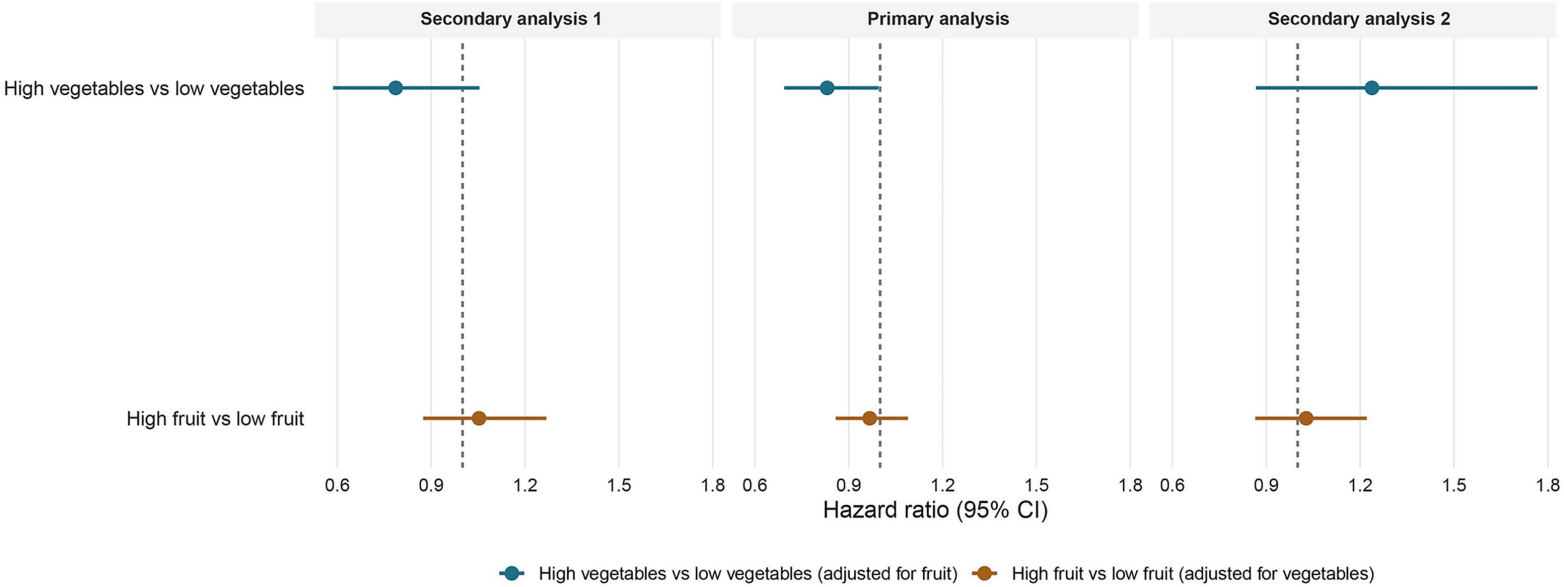
Associations of baseline fruit and vegetable consumption frequency with tooth-loss transitions across the three analysis cohorts. Points indicate hazard ratios and horizontal bars indicate 95% confidence intervals from the primary adjustment model.

Baseline characteristics of the primary cohort, defined as participants with 1–19 baseline teeth, are shown in Table 1. Participants who reported more frequent vegetable consumption were generally younger than those with less frequent vegetable consumption, with median ages of 83 and 86 years, respectively. They were also more often married, more likely to live in city or town settings, and more likely to report regular exercise. Denture use was more common among participants with more frequent vegetable consumption, whereas low body mass index and multiple ADL limitations were more frequent among those with less frequent vegetable consumption. Other baseline differences were mixed (Figure 2).

**Table 1:** Baseline characteristics of participants with 1–19 baseline teeth, by baseline vegetable consumption frequency.

Variable	Overall *n* = 3,919	Lower vegetable consumption frequency *n* = 373	Higher vegetable consumption frequency *n* = 3,546	*p* value
Age, years	83 (75, 90)	86 (79, 92)	83 (75, 90)	<0.001
Sex				**0.3**
Female	2,179 (56%)	217 (58%)	1,962 (55%)	
Male	1,740 (44%)	156 (42%)	1,584 (45%)	
Education, years				**0.023**
0	2,354 (60%)	247 (66%)	2,107 (59%)	
1–6	1,187 (30%)	104 (28%)	1,083 (31%)	
≥7	375 (9.6%)	22 (5.9%)	353 (10.0%)	
Marital status				**<0.001**
Married	1,466 (37%)	100 (27%)	1,366 (39%)	
Others	2,453 (63%)	273 (73%)	2,180 (61%)	
Residence				**0.011**
City/town	1,460 (37%)	116 (31%)	1,344 (38%)	
Rural	2,459 (63%)	257 (69%)	2,202 (62%)	
Current smoking, yes	714 (18%)	48 (13%)	666 (19%)	0.006
Current alcohol drinking, yes	735 (19%)	57 (15%)	678 (19%)	0.082
Regular exercise				**0.005**
No	2,676 (68%)	279 (75%)	2,397 (68%)	
Yes	1,243 (32%)	94 (25%)	1,149 (32%)	
Sleep duration				**0.9**
<7 h	1,080 (28%)	109 (29%)	971 (27%)	
7–9 h	1,906 (49%)	175 (47%)	1,731 (49%)	
>9 h	924 (24%)	88 (24%)	836 (24%)	
Body mass index category, kg/m^2^				**0.002**
<18.5	1,161 (30%)	141 (38%)	1,020 (29%)	
18.5–23.9	2,177 (56%)	194 (52%)	1,983 (56%)	
24.0–27.9	457 (12%)	31 (8.3%)	426 (12%)	
≥28	91 (2.3%)	4 (1.1%)	87 (2.5%)	
ADL limitations (number of impaired activities)				**<0.001**
0	3,580 (91%)	331 (89%)	3,249 (92%)	
1	174 (4.4%)	12 (3.2%)	162 (4.6%)	
≥2	165 (4.2%)	30 (8.0%)	135 (3.8%)	
Denture use				**<0.001**
No	2,981 (76%)	314 (84%)	2,667 (75%)	
Yes	938 (24%)	59 (16%)	879 (25%)	
Hypertension				**0.5**
No	3,009 (77%)	295 (79%)	2,714 (77%)	
Yes	837 (21%)	71 (19%)	766 (22%)	
Diabetes				**0.8**
No	3,759 (96%)	360 (97%)	3,399 (96%)	
Yes	100 (2.6%)	8 (2.1%)	92 (2.6%)	
Heart disease				**0.3**
No	3,508 (90%)	343 (92%)	3,165 (89%)	
Yes	352 (9.0%)	26 (7.0%)	326 (9.2%)	
Stroke				**0.2**
No	3,674 (94%)	346 (93%)	3,328 (94%)	
Yes	193 (4.9%)	24 (6.4%)	169 (4.8%)	

Values are median (Q1, Q3) or *n* (%). *p* values were calculated using the Kruskal-Wallis test for continuous variables and Pearson’s chi-square test for categorical variables. Categories for missing covariate data were included in model fitting but are not displayed in this table. Bold *p* values indicate *p* < 0.05.

### 3.3 Incident edentulism in participants with 1–19 baseline teeth

Associations in participants with 1–19 baseline teeth are presented in Table 2; the primary-model estimates across all three dentition strata are summarised in Figure 2. For target-population interpretation, we first examined the full baseline-eligible cohort in which death was handled as a competing outcome. In this broader risk set, more frequent vegetable consumption showed an attenuated inverse association with incident edentulism (HR 0.88, 95% CI 0.73–1.04), and this estimate was materially unchanged after additional adjustment for self-rated local economic status (f34) (HR 0.88, 95% CI 0.74–1.04). Fruit consumption was close to the null in this framework (HR 1.00, 95% CI 0.88–1.12), and the corresponding cause-specific death estimate for vegetable consumption was also close to the null (HR 0.94, 95% CI 0.84–1.05).

**Table 2:** Associations of baseline fruit and vegetable consumption frequency with incident edentulism and competing death among participants with 1–19 baseline teeth.

Outcome framework	Model	Higher vs. lower fruit consumption frequency		Higher vs. lower vegetable consumption frequency	
		HR (95% CI)	*p* value	HR (95% CI)	*p* value
Full baseline-eligible cohort: cause-specific incident edentulism	Extended model	1.00 (0.88, 1.12)	0.940	0.88 (0.73, 1.04)	0.138
	Extended model + self-rated local economic status	1.01 (0.89, 1.13)	0.917	0.88 (0.74, 1.04)	0.141
Full baseline-eligible cohort: cause-specific death	Extended model	0.93 (0.87, 1.01)	0.078	0.94 (0.84, 1.05)	0.255
	Extended model + self-rated local economic status	0.94 (0.87, 1.01)	0.101	0.94 (0.84, 1.05)	0.270
Observed-outcome sample: incident edentulism	Extended model	0.97 (0.86, 1.09)	0.598	0.83 (0.69, 0.99)	0.040
	Extended model + self-rated local economic status	0.98 (0.87, 1.11)	0.748	0.83 (0.69, 1.00)	0.044

Values are hazard ratios with 95% confidence intervals. Fruit and vegetable consumption frequency were mutually adjusted in all models. The extended model adjusted for age, sex, education, marital status, residence, smoking, alcohol drinking, regular exercise, sleep duration, activities of daily living limitations, body mass index category, baseline natural teeth count, denture use, hypertension, diabetes, heart disease, and stroke. The full baseline-eligible cohort included participants regardless of later tooth-count observation and handled death as a competing outcome; the observed-outcome sample required observed follow-up tooth-count data. The socioeconomic model additionally adjusted for self-rated local economic status.

**Figure 3:**
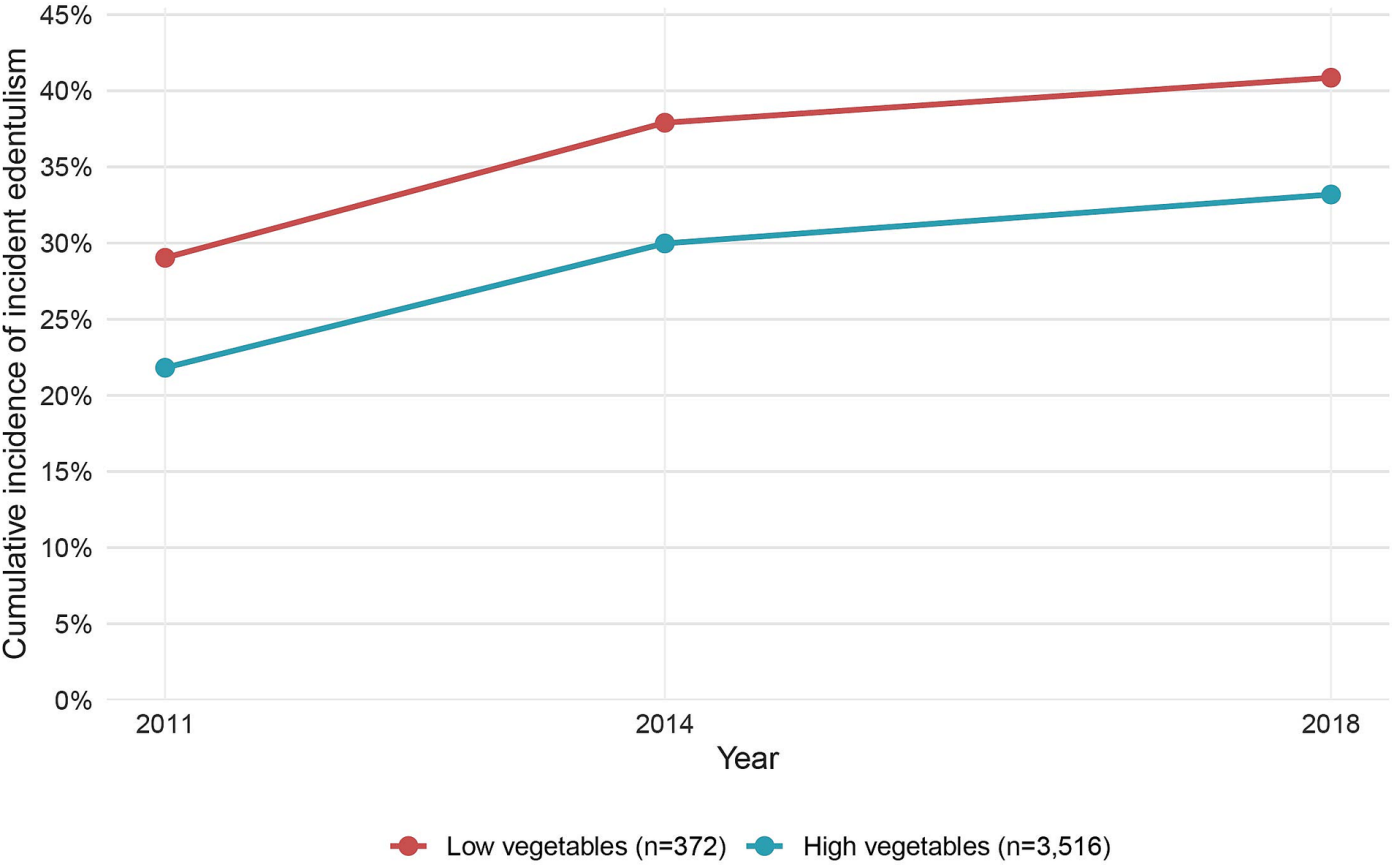
Crude cumulative incidence of incident edentulism by baseline vegetable consumption frequency in the primary cohort. Estimates were derived from the unweighted person-period sample after application of the sequential follow-up rule. Low vegetables, *n* = 372; high vegetables, *n* = 3,516.

In the observed-outcome sample with follow-up tooth-count data, more frequent fruit consumption was not associated with incident edentulism in the extended model (HR 0.97, 95% CI 0.86–1.09), whereas more frequent vegetable consumption was associated with a lower hazard of incident edentulism (HR 0.83, 95% CI 0.69–0.99). This observed-outcome estimate was stronger than the estimate from the full baseline-eligible cohort and should be interpreted in that context. Crude cumulative incidence of incident edentulism was consistently lower among participants with higher baseline vegetable consumption frequency, reaching 21.8% versus 29.0% by 2011, 30.0% versus 37.9% by 2014, and 33.2% versus 40.9% by 2018 (Figure 3 and Supplementary Table S16).

### 3.4 Secondary analyses in other baseline dentition strata

Results in the other predefined baseline dentition strata were weaker and less consistent than those observed in the primary analysis, as shown in Table 3. Because the secondary analyses evaluated transition to fewer than 20 teeth rather than incident edentulism, these estimates were used to assess consistency across stages rather than for direct comparison with the primary hazard ratio. In participants with 20–24 baseline teeth, more frequent vegetable consumption showed a directionally inverse but imprecise association in the primary model (HR 0.79, 95% CI 0.59–1.05), whereas fruit consumption frequency remained close to the null (HR 1.05, 95% CI 0.87–1.27).

**Table 3:** Associations of baseline fruit and vegetable consumption frequency with tooth-loss transitions in the two secondary analyses.

Exposure	Minimal model		Primary model		Extended model	
	HR (95% CI)	*p*	HR (95% CI)	*p*	HR (95% CI)	*p*
Participants with 20–24 baseline teeth						
Higher vs. lower fruit consumption frequency (adjusted for vegetable consumption frequency)	1.01 (0.85, 1.22)	0.884	1.05 (0.87, 1.27)	0.584	1.06 (0.88, 1.28)	0.525
Higher vs. lower vegetable consumption frequency (adjusted for fruit consumption frequency)	0.84 (0.63, 1.13)	0.244	0.79 (0.59, 1.05)	0.108	0.80 (0.60, 1.08)	0.148
Participants with 25–32 baseline teeth						
Higher vs. lower fruit consumption frequency (adjusted for vegetable consumption frequency)	0.91 (0.77, 1.07)	0.243	1.03 (0.86, 1.22)	0.758	1.03 (0.87, 1.23)	0.725
Higher vs. lower vegetable consumption frequency (adjusted for fruit consumption frequency)	1.11 (0.78, 1.58)	0.548	1.24 (0.87, 1.77)	0.240	1.24 (0.87, 1.76)	0.235

HR, hazard ratio; CI, confidence interval. Model specifications followed the minimal, primary, and extended adjustment sets described in Methods section 2.6. In both secondary analyses, the outcome was incident loss of functional dentition, defined as fewer than 20 natural teeth at follow-up. Because the secondary analyses used a different endpoint from the primary incident-edentulism analysis, estimates should be interpreted as stage-specific transition estimates rather than directly compared across strata.

In participants with 25–32 baseline teeth, neither fruit consumption frequency nor vegetable consumption frequency showed evidence of an inverse association. The corresponding primary-model estimates were 1.03 (95% CI 0.86–1.22) for fruit consumption frequency and 1.24 (95% CI 0.87–1.77) for vegetable consumption frequency; the corresponding extended-model estimates were 1.03 (95% CI 0.87–1.23) and 1.24 (95% CI 0.87–1.76), respectively. These patterns suggest that the clearest association was concentrated in participants who had already experienced substantial tooth loss but were not yet edentulous at baseline.

### 3.5 Sensitivity and exploratory analyses

Additional sensitivity and supplementary diagnostic analyses are summarised in Supplementary Tables S7–S22 and Supplementary Figures S1–S3. Functional-form sensitivity analyses for baseline tooth count yielded nearly identical estimates in the primary analysis when baseline tooth count was modelled as a factor rather than a linear term. Single-component models showed the same general pattern, with an inverse association for vegetable consumption frequency but not for fruit consumption frequency. Vegetable estimates were stronger in the time-updated and cumulative-average analyses, a pattern interpreted cautiously because follow-up diet may partly reflect evolving oral function, frailty, or preterminal decline rather than a dose-response relation. Additional adjustment for Parkinson’s disease and epilepsy did not materially alter the findings. Exploratory joint exposure analyses again suggested that the more consistent association was linked to higher vegetable consumption frequency rather than higher fruit consumption frequency alone. In the primary cohort, the high-fruit and low-vegetable subgroup comprised only 33 participants (0.8%), which limited the precision of that contrast. Exploratory denture-stratified analyses suggested a similar direction of association among participants with and without dentures, and the formal interaction test did not indicate clear effect modification (*p* for interaction = 0.537; Supplementary Table S12).

Missingness in baseline covariates was low in the primary cohort, and complete-case estimates were closely aligned with the main extended model. Analyses using the original ordered response categories supported an inverse trend for vegetable consumption frequency but not for fruit consumption frequency. Interval-specific follow-up status showed that death before interval end without observed tooth count became more common after the first interval, whereas missing tooth count or censoring after cohort entry was broadly similar across vegetable consumption groups within later intervals. We found no clear evidence that the fruit or vegetable associations varied across follow-up intervals. In death-related sensitivity analyses, the inverse association for vegetable consumption frequency was attenuated when a composite endpoint incorporating death was considered. Inclusion-weighting analyses addressing differential entry into the primary analysis also attenuated the vegetable estimate.

## 4 Discussion

In this prospective analysis of older Chinese adults, higher vegetable consumption frequency showed only a weak inverse association with incident edentulism among participants with 1–19 baseline teeth when the full baseline-eligible cohort and competing death were considered, whereas fruit consumption remained near the null. A stronger vegetable estimate was observed in the restricted observed-outcome sample with follow-up tooth-count data, but this difference indicates sensitivity to selection and competing survival rather than robust evidence of an independent protective association. Comparable associations were not evident in the 20–24 or 25–32 baseline-teeth strata. These findings support a cautious interpretation of a stage-specific observational association.

This pattern is consistent with broader evidence that oral health and diet are closely linked in later life. Edentulism remains an important endpoint of cumulative oral disadvantage in older adults (15), and earlier studies have shown that greater tooth loss is associated with less frequent fruit and vegetable consumption and less favourable nutrient intake profiles (8, 16). Retention of natural teeth has likewise been associated with greater dietary diversity and better nutritional status in older adults (9, 17). Our study extends this literature by examining the prospective direction of this relationship in a Chinese longitudinal cohort and by focusing on clinically discrete tooth-loss transitions rather than tooth count alone (12).

The Chinese context also matters for interpretation. Dental service utilisation among middle-aged and older adults in China has remained low and socially patterned, with education, income, and related socioeconomic factors influencing whether and how often care is used (18, 19). National survey evidence further shows clear urban-rural and educational gradients in tooth retention among older Chinese adults (20). Among older Chinese adults with edentulism, denture use also differs substantially between urban and rural settings, and these differences are partly explained by education, income, and chronic disease burden (21). More broadly, recent life-course evidence indicates marked socioeconomic inequalities in oral health among Chinese older adults (22). In this setting, baseline vegetable consumption frequency may be especially likely to reflect broader social, behavioural, and oral-functional advantage rather than an isolated dietary effect, and the present findings should therefore be interpreted within this specific historical and healthcare context rather than assumed to represent a directly transportable causal effect.

This pattern is clinically plausible. Once dentition has already declined to 1–19 teeth, progression to edentulism becomes a more discrete and clinically meaningful transition than movement across higher tooth-count ranges. The 20-tooth threshold remains a pragmatic benchmark for functional dentition in older adults (3), and retention of 20 or more natural teeth has been associated with better survival, health, and quality of life in later life (4, 5). Tooth loss has also been discussed as a marker of broader vulnerability in ageing populations (23, 24), which may help explain why diet-related associations in our study were most evident after dentition had already become substantially compromised.

The more consistent association for vegetable consumption frequency than for fruit consumption frequency also warrants cautious interpretation. Fruit and vegetable intake has been linked to periodontal health and oral health-related quality of life (25, 26), whereas poor oral status in later life often coexists with nutritional vulnerability and adverse general health (27, 28). In this context, higher vegetable consumption frequency in our study may reflect broader diet quality, behavioural resilience, and social or functional advantage that was only partly captured by the measured covariates. This interpretation is also compatible with the common risk factor perspective, in which diet, chronic disease risk, and oral health arise from shared upstream determinants (29).

Denture-stratified analyses did not show clear effect modification by baseline denture use, and the stratum-specific estimates were imprecise. This pattern is clinically plausible because dentures may partly compensate for chewing impairment, yet prosthetic rehabilitation alone does not consistently restore dietary behaviour or nutritional adequacy (13). Evidence from review and interventional studies further suggests that dietary improvement is more likely when oral rehabilitation is combined with dietary advice rather than delivered in isolation (30–32).

The findings in participants with 25–32 baseline teeth help define the boundary of interpretation. In this stratum, transition to fewer than 20 teeth likely reflects a longer and more heterogeneous pathway, in which diet is only one of several influences. Evidence on shortened dental arch therapy suggests that tooth number, oral function, and nutrition do not map onto one another in a simple linear way (33). Oral health in later life also affects quality of life and frailty through pathways that extend beyond tooth count alone (34, 35).

This study has several strengths. We used a large longitudinal cohort with repeated follow-up, predefined clinically meaningful dentition strata, and a person-period framework aligned with the timing of observed tooth-count data. A further strength was the use of complementary approaches to selection and competing survival, including the full baseline-eligible cohort competing-outcome analysis, inclusion weighting, and death-related endpoints. The attenuation of the vegetable estimate across these approaches supported a conservative interpretation of the observed-outcome association.

From a nutritional perspective, the practical implication is limited but relevant. Our data do not support using fruit or vegetable consumption frequency alone as stand-alone markers of subsequent edentulism risk, nor do they justify causal claims about preventing edentulism. Instead, they support a more integrated view of late-life oral ageing in which dietary behaviour, baseline dentition status, and broader health vulnerability are considered together. Oral health has increasingly been positioned as a core component of healthy ageing rather than a separate domain (6, 36), and it remains an important determinant of quality of life in older adults (34). Longitudinal evidence also suggests that tooth loss, diet quality, and other ageing-related outcomes may be intertwined over time (37). In this context, less frequent vegetable consumption in an older adult with already reduced dentition may be interpreted as a marker of broader vulnerability that warrants attention alongside oral rehabilitation, dietary support, and general risk-factor management.

Several limitations should be considered alongside these strengths. Both the exposure and the outcome were self-reported, and the dietary measures were coarse. Grouping the “almost every day except in winter” response with “almost every day” may have obscured seasonal variation in vegetable intake, and the data did not capture portion size, preparation method, overall dietary pattern, or nutrient composition. We also lacked key oral-health variables, including periodontal status, caries history, oral hygiene practises, chewing ability, occlusal support, dental service use, denture quality, and reasons for extraction. Because participants who were edentulous at baseline were not eligible, the findings do not generalise to older adults who had already lost all natural teeth at cohort entry. Although the prospective design reduces simple reverse-causation concerns, it cannot fully disentangle the bidirectional relationship between diet and oral health in later life. The additional sensitivity analyses, including complete-case, ordered-category, interval-stability, and socioeconomic-adjustment analyses, supported the overall pattern of the findings but do not remove the likelihood of residual confounding. We also examined two dietary exposures across three dentition strata and multiple supplementary analyses without formal adjustment for multiplicity, so the nominally significant observed-outcome vegetable estimate should be regarded as exploratory. Most importantly, the attenuation seen in the inclusion-weighted, death-related, and full baseline-eligible cohort competing-outcome analyses suggests that differential survival and analytic inclusion contributed materially to the observed inverse association for vegetable consumption frequency.

## 5 Conclusion

Among older Chinese adults with 1–19 baseline natural teeth, higher vegetable consumption frequency showed a weak inverse association with incident edentulism in the full baseline-eligible cohort when death was treated as a competing outcome, whereas fruit consumption frequency remained near null. The stronger inverse association observed among participants with follow-up tooth counts should be interpreted cautiously, as it may partly reflect selection and differential survival; the data do not establish an independent protective effect of vegetable consumption frequency.

## Data Availability

The data analyzed in this study are available from the Chinese Longitudinal Healthy Longevity Survey through the Peking University Open Research Data Platform (doi: 10.18170/DVN/WBO7LK), subject to registration and the platform’s terms of use. Further inquiries can be directed to the corresponding authors.
