# Supplementary material for: Fruit and vegetable consumption frequency and tooth-loss transitions across baseline dentition strata in older Chinese adults: a prospective cohort study
Source: Front Nutr. 2026 Jul 17;13:1829512. doi: 10.3389/fnut.2026.1829512 (PMC13423628; doi:10.3389/fnut.2026.1829512)

**Supplementary Appendix**

**Supplementary Methods**

*eMethods 1. Data processing and analytic sample construction*

Analytic files were merged by participant identifier after confirmation of the required variables. We then applied prespecified cleaning rules for baseline tooth count, denture use, fruit consumption frequency, vegetable consumption frequency, and baseline covariates before defining the analysis cohorts. Participants first had to be aged 65 years or older at baseline and to have valid information on baseline natural teeth count.

Participants were then classified into three predefined baseline dentition strata: 1–19, 20–24, and 25–32 baseline teeth. The primary analysis included participants with 1–19 baseline teeth, whereas the two secondary analyses included participants with 20–24 and 25–32 baseline teeth, respectively. For the observed-outcome analyses, participants additionally required non-missing baseline fruit and vegetable consumption frequency data and at least one observed follow-up tooth count after baseline. The full baseline-eligible cohort competing-outcome analysis in the 1–19-teeth stratum required non-missing baseline fruit and vegetable consumption frequency data but did not require an observed follow-up tooth count.

*eMethods 2. Exposure definitions*

Self-reported fruit and vegetable consumption frequency was assessed using standard CLHLS questions. The primary exposure specification used baseline fruit consumption frequency and baseline vegetable consumption frequency coded as higher versus lower frequency. Fruit consumption frequency was classified as higher for responses of almost every day or quite often and lower for responses of occasionally or rarely or never. Vegetable consumption frequency was classified as higher for the two most frequent response categories, “almost every day” and “almost every day except in winter,” and as lower when participants reported eating vegetables occasionally or rarely or never. This binary grouping followed the CLHLS response structure; in particular, the “almost every day except in winter” category may not represent the same year-round intake pattern as “almost every day.” The principal models included fruit and vegetable consumption frequency simultaneously to estimate mutually adjusted associations.

Baseline exposure was retained as the primary specification because the main question concerned whether dietary frequency reported at cohort entry was associated with subsequent tooth-loss transitions. In older adults with declining dentition, updating diet during follow-up may partly capture downstream changes related to tooth-loss progression, chewing difficulty, frailty, or preterminal decline. Time-updated and cumulative-average exposure definitions were therefore treated as sensitivity analyses rather than primary specifications. Accordingly, stronger estimates in time-updated or cumulative-average analyses were interpreted as compatible with changes in diet following oral or functional decline rather than as dose-response evidence.

To assess whether the binary exposure definition discarded meaningful information from the original response categories, additional analyses in the primary cohort used the original ordered fruit and vegetable frequency categories. These analyses included an ordinal trend model and a four-level factor model. Because the questionnaire responses were ordinal categories rather than continuous intake measures, ordered-category and factor specifications were used rather than spline-based dose–response modelling. Exploratory joint exposure models combined baseline fruit and vegetable consumption frequency into four categories: lower fruit plus lower vegetables, higher fruit plus lower vegetables, lower fruit plus higher vegetables, and higher fruit plus higher vegetables.

*eMethods 3. Outcome definitions and person-period data*

Self-reported natural teeth counts were available at the 2008, 2011, 2014, and 2018 waves. In the primary analysis, the event of interest was incident edentulism, defined as 0 natural teeth at follow-up. In the two secondary analyses, the event of interest was loss of functional dentition, defined as fewer than 20 natural teeth at follow-up.

Person-period datasets were created across the intervals 2008–2011, 2011–2014, and 2014–2018. Participants contributed interval-specific observations only when the outcome at the end of that interval was observed. If end-of-interval tooth-count data were missing, participants were censored at the start of that interval and did not contribute subsequent intervals. For analyses using alternative exposure definitions, once an interval lacked the required interval-specific exposure or end-of-interval outcome information, later intervals were not carried forward.

*eMethods 4. Covariates, missing data, and baseline tooth-count parameterization*

Baseline covariates included age, sex, education, marital status, residence, current smoking, current alcohol drinking, regular exercise, sleep duration, body mass index category, activities of daily living limitations, denture use, hypertension, diabetes, heart disease, and stroke. Parkinson’s disease and epilepsy were added in an additional sensitivity analysis. Baseline natural teeth count was adjusted for within each baseline dentition stratum.

Categorical covariates were modelled with explicit missing categories to preserve the analytic sample and avoid excluding participants with otherwise usable outcome and exposure data. To make this strategy transparent, we additionally reported the amount of missingness in the original baseline covariate variables before missing-category coding. We also performed a complete-case sensitivity analysis in the primary cohort using the same extended covariate set as the main fully adjusted model.

Baseline natural teeth count was modelled linearly in the primary models within each baseline dentition stratum. Sensitivity analyses reparameterised baseline tooth count using factor coding to assess whether the principal findings depended on the assumed linear form.

*eMethods 5. Primary, parallel, and exploratory models*

Discrete-time complementary log-log regression models were fitted with interval indicators and an offset for log interval length. The minimal model adjusted for age and sex. The primary model additionally adjusted for education, marital status, residence, smoking, drinking, exercise, sleep duration, activities of daily living limitations, body mass index category, baseline tooth count, and denture use. The extended model further adjusted for hypertension, diabetes, heart disease, and stroke. Robust standard errors were clustered by participant.

Parallel analyses were conducted in participants with 20–24 baseline teeth and in participants with 25–32 baseline teeth using the same modelling framework. Supporting analyses examined single-component models, alternative exposure definitions, additional adjustment for Parkinson’s disease and epilepsy, exploratory joint exposure models, denture-stratified analyses, death-related endpoints, inclusion weighting, complete-case analysis, original ordered exposure categories, interval-specific follow-up status, and interval-stability and model-fit diagnostics in the primary cohort.

*eMethods 6. Death-related sensitivity analyses*

Death-related sensitivity analyses were performed in each baseline dentition stratum. Composite endpoint models treated either the tooth-loss event or death as the event of interest, whereas death-only models treated death as the endpoint. These analyses were intended to assess the extent to which mortality during follow-up might alter interpretation of the primary tooth-loss associations.

*eMethods 7. Inclusion-weighting sensitivity analysis and cumulative incidence*

Because entry into the primary analysis required observable follow-up tooth-count data, we conducted an additional inclusion-weighting sensitivity analysis for this cohort. A baseline-eligible subset was first defined among participants with 1–19 baseline teeth and non-missing baseline fruit and vegetable consumption frequency data. We then modelled the probability of entering the primary analytic cohort, defined by non-missing baseline fruit and vegetable consumption frequency data and at least one observed follow-up natural teeth count, using baseline sociodemographic, behavioural, functional, anthropometric, oral-health, chronic-disease, and exposure variables. This weighting analysis targeted entry into the pre-person-period analytic cohort rather than the final regression sample after application of the sequential follow-up rule.

Stabilized inclusion weights were calculated using the observed probability of inclusion in the numerator and the predicted probability of inclusion in the denominator. We additionally examined weights truncated at the 1st and 99th percentiles. The primary analysis model was then repeated using these inclusion weights. Absolute cumulative incidence by baseline vegetable consumption frequency was also calculated from the unweighted person-period sample for descriptive interpretation.

Table S2 was based on the full baseline 1–19 teeth stratum, whereas Table S14 was restricted to the baseline-eligible subset with non-missing baseline fruit and vegetable consumption frequency data. Accordingly, the denominators in Tables S2 and S14 differ slightly.

*eMethods 8. Additional primary-cohort sensitivity and diagnostic analyses*

Several additional analyses were conducted in the primary cohort to address missing data, exposure granularity, selective follow-up, and model diagnostics. First, missingness was summarised for the original baseline covariate variables before explicit missing-category coding, and the extended model was repeated in participants with complete baseline covariate data. Second, the original ordered fruit and vegetable frequency categories were examined using an ordinal trend model and a four-level factor model. Third, interval-specific participant status was tabulated by baseline vegetable consumption frequency, including incident edentulism, death before interval end without observed tooth count, missing tooth count or censoring, and observed non-event with continued follow-up. Fourth, interval stability of the fruit and vegetable associations was assessed by robust Wald tests for exposure-by-interval interaction terms in the extended model. Overall model fit was summarised by comparing the observed event proportion with the mean predicted probability and by calculating the Brier score in the final person-period regression sample.

*eMethods 9. Full baseline-eligible cohort competing-outcome analysis and additional socioeconomic adjustment*

To evaluate the potential influence of restricting the observed-outcome analysis to participants with observed follow-up tooth counts, we conducted an additional analysis in the full baseline-eligible cohort within the 1–19 baseline-teeth stratum. This cohort included all participants with 1–19 baseline natural teeth and non-missing baseline fruit and vegetable consumption frequency data, regardless of whether a follow-up tooth count was later observed.

Within this full baseline-eligible cohort, interval-specific participant status was classified as incident edentulism, death before interval end without observed tooth count, missing tooth count or censored, or observed non-event with continued follow-up. Cause-specific incident edentulism and cause-specific death were then modelled separately using discrete-time complementary log-log regression. For the corresponding cause-specific model, competing-event rows were retained in the risk set and coded as non-events. We fitted the same extended adjustment model used in the primary analysis and then repeated the full baseline-eligible cohort models with additional adjustment for self-rated local economic status (f34).

**Supplementary Results**

*S1. Selection before entry into the primary analyses*

Selection patterns before entry into the primary analyses are shown in Tables S1 to S3. Across all three dentition strata, participants excluded before the primary analyses were generally older and exhibited less favourable baseline profiles than those retained. The contrast was most pronounced in the primary analysis, where excluded participants had markedly older age distributions, less exercise, more ADL limitation, lower baseline tooth counts, and lower prevalence of denture use. Table S2 describes selection within the full baseline 1–19 teeth stratum (n = 7,742), whereas the inclusion-weighting analysis reported in Table S14 was restricted to the baseline-eligible subset with non-missing baseline fruit and vegetable consumption frequency data (n = 7,740). Accordingly, the corresponding denominators differ slightly.

*S2. Baseline characteristics in the two secondary cohorts*

Tables S4 and S5 summarise baseline characteristics by baseline vegetable consumption frequency in participants with 20–24 baseline teeth and in participants with 25–32 baseline teeth. In participants with 20–24 baseline teeth, those with higher vegetable consumption frequency were somewhat less likely to have no formal education and slightly more likely to use dentures, but group differences were otherwise modest. In participants with 25–32 baseline teeth, higher vegetable consumption frequency was associated with a younger age profile, more education, and more regular exercise.

*S3. Distribution of baseline joint fruit and vegetable consumption frequency categories*

Table S6 shows the distribution of the four baseline joint fruit and vegetable consumption frequency categories across the three analysis cohorts. In all three baseline dentition strata, the high-fruit and low-vegetable category was sparse, accounting for fewer than 2% of participants. By contrast, the two categories characterised by higher vegetable consumption frequency comprised the majority of participants in each cohort. These data help explain why joint exposure analyses were treated as exploratory rather than primary. The baseline distributions of the original fruit and vegetable frequency categories are shown in Figure S1 and further illustrate that vegetable consumption frequency was concentrated in the more frequent categories, whereas fruit consumption frequency was more broadly distributed across response levels.

*S4. Functional-form and single-component sensitivity analyses*

Table S7 indicates that changing the functional form of baseline tooth count did not materially alter the primary analysis results. The primary-model estimate for higher vegetable consumption frequency remained essentially unchanged when baseline tooth count was entered as a factor rather than a linear term. Table S8 shows that the single-component models yielded the same broad pattern, with an inverse association for vegetable consumption frequency in the primary analysis and no comparable association for fruit consumption frequency.

*S5. Alternative exposure definitions and additional adjustment*

Table S9 presents sensitivity analyses using baseline, time-updated, and cumulative-average exposure definitions. In the primary analysis, vegetable estimates were stronger in the time-updated and cumulative-average analyses, whereas fruit estimates remained close to the null. Because follow-up diet may partly reflect evolving oral function, frailty, or preterminal decline, these estimates were not interpreted as dose-response evidence. Table S10 shows that additional adjustment for Parkinson’s disease and epilepsy did not materially change the pattern of results.

*S6. Exploratory joint-exposure, denture-stratified, and death-related analyses*

Table S11 presents exploratory joint fruit and vegetable models. In the primary analysis, both categories characterised by higher vegetable consumption frequency were associated with lower hazards of incident edentulism, whereas the high-fruit and low-vegetable category remained imprecise because of sparse cell counts. Figure S3 provides a graphical summary of these exploratory joint-exposure estimates in the primary analysis. Table S12 shows exploratory denture-stratified estimates for vegetable consumption frequency in the primary analysis. Although the point estimate was numerically lower among denture users than among non-users, the formal interaction test did not indicate clear evidence of effect modification (P for interaction = 0.537). Death-related sensitivity analyses are summarised in Table S13. In the primary analysis, the inverse association for higher vegetable consumption frequency was attenuated in the composite endpoint analysis and was not evident in the death-only analysis. Taken together, these findings indicate that the association for vegetable consumption frequency was sensitive to selective follow-up and competing survival.

*S7. Inclusion-weighting and absolute risk analyses*

Table S14 summarises the inclusion-weighting diagnostics for the primary analysis. The denominator in Table S14 differs slightly from that in Table S2 because Table S2 was based on the full baseline 1–19 teeth stratum (n = 7,742), whereas Table S14 was restricted to the baseline-eligible subset with non-missing baseline fruit and vegetable consumption frequency data (n = 7,740). Among these 7,740 baseline-eligible participants, 3,919 entered the primary analytic cohort with non-missing baseline fruit and vegetable consumption frequency data and at least one follow-up natural teeth count, corresponding to an observed inclusion probability of 0.506. The final person-period regression sample was slightly smaller (n = 3,888) after application of the sequential follow-up rule. Predicted inclusion probability was higher among included participants than among excluded participants, and the stabilised inclusion weights were centred close to 1.0, although the upper tail extended beyond 3 before truncation.

Table S15 compares the unweighted, inclusion-weighted, and truncated inclusion-weighted primary models. After weighting, the point estimate for higher vegetable consumption frequency moved towards the null, from 0.83 in the unweighted primary model to 0.88 with full inclusion weighting and 0.85 after truncation. Fruit consumption frequency remained close to the null in all three versions of the model. Table S16 and Figure S2 show that crude cumulative incidence of edentulism remained consistently lower among participants with higher baseline vegetable consumption frequency across follow-up, despite the attenuation seen after weighting.

*S8. Missing covariate data and complete-case sensitivity in the primary cohort*

Missingness in baseline covariates was low in the primary cohort before explicit missing-category coding. The highest level of missingness was observed for Parkinson’s disease (2.99%), followed by hypertension (1.86%), diabetes (1.53%), heart disease (1.51%), stroke (1.33%), body mass index category (0.84%), epilepsy (0.77%), sleep duration (0.23%), and education (0.08%) (Table S17). No missing data were present for age, sex, marital status, residence, smoking, drinking, exercise, activities of daily living limitations, denture use, or baseline natural teeth count.

Complete-case sensitivity analysis yielded estimates that were closely aligned with the main extended model. In the final person-period sample, the vegetable estimate was 0.83 (95% CI 0.69–0.99) in the extended model with explicit missing categories and 0.82 (95% CI 0.68–0.99) in the complete-case analysis. The fruit estimate remained close to the null in both analyses (Table S18).

*S9. Analyses using the original ordered exposure categories*

Analyses using the original ordered response categories supported the main interpretation of a vegetable-related signal and a near-null fruit association in the primary cohort. In the ordinal trend model, each one-category higher level of fruit consumption frequency was not associated with incident edentulism (HR 0.98, 95% CI 0.92–1.05), whereas each one-category higher level of vegetable consumption frequency was associated with a lower hazard of incident edentulism (HR 0.89, 95% CI 0.83–0.96) (Table S19). In the four-level factor model, the fruit estimates remained close to the null. Vegetable estimates were directionally lower for the more frequent categories, although confidence intervals were wider because the rarely-or-never vegetable group was small.

*S10. Interval-specific follow-up status, interval stability, and model fit*

Interval-specific follow-up status in the primary analytic cohort is shown in Table S20. The absence of deaths before interval end in 2008–2011 in Table S20 reflects construction of the observed-outcome analytic cohort rather than absence of mortality in the baseline-eligible population; participants who died before the 2011 wave without an observed tooth count did not enter the observed-outcome cohort and are shown in the full baseline-eligible cohort in Table S22. Among participants with lower baseline vegetable consumption frequency, 108 of 373 developed incident edentulism during 2008–2011, 33 of 264 during 2011–2014, and 11 of 118 during 2014–2018. Among participants with higher baseline vegetable consumption frequency, the corresponding counts were 767 of 3,546, 287 of 2,749, and 113 of 1,453. Death before interval end without observed tooth count became more common after the first interval, particularly in the 2011–2014 and 2014–2018 intervals. Missing tooth count or censoring after entry showed broadly similar proportions across vegetable consumption groups within later intervals.

We found no clear evidence that the associations of baseline fruit or vegetable consumption frequency varied across follow-up intervals. Robust Wald tests for exposure-by-interval interaction terms gave P=0.527 for fruit frequency and P=0.699 for vegetable frequency (Table S21). In the same extended model, the overall observed event proportion was 0.2028 and the overall mean predicted probability was 0.2027, with a Brier score of 0.1514.

*S11. Full baseline-eligible cohort competing-outcome analysis and additional socioeconomic adjustment*

Table S22 summarises interval-specific participant status in the full baseline-eligible cohort with 1–19 baseline teeth and non-missing baseline fruit and vegetable consumption frequency data (n=7,740). This table shows that substantial numbers of participants were not represented in the observed-outcome sample because of death before interval end without observed tooth count or missing tooth count/censoring, particularly after the first interval.

The corresponding model summary is presented in the main text Table 2. In the full baseline-eligible cohort, the cause-specific incident edentulism estimate for higher vegetable consumption frequency was 0.88 (95% CI 0.73–1.04), compared with 0.83 (95% CI 0.69–0.99) in the observed-outcome sample. Additional adjustment for self-rated local economic status (f34) did not materially alter this result. These analyses indicate that the inverse association observed in the restricted observed-outcome sample was attenuated when the full baseline-eligible cohort and competing survival were taken into account.

**Supplementary Tables**

**Table S1. Baseline characteristics of participants included in and excluded from the secondary analysis in participants with 20–24 baseline teeth.**

| **Variable** | **Included in analysis N = 754** | **Excluded before analysis N = 384** | ***P* value** |
| --- | --- | --- | --- |
| Age, years | 73 (69, 81) | 80 (71, 90) | <0.001 |
| **Sex** |  |  | **0.11** |
| Female | 365 (48%) | 166 (43%) |  |
| Male | 389 (52%) | 218 (57%) |  |
| **Education, years** |  |  | **0.11** |
| 0 | 324 (43%) | 187 (49%) |  |
| 1-6 | 306 (41%) | 132 (34%) |  |
| ≥7 | 124 (16%) | 65 (17%) |  |
| **Marital status** |  |  | **<0.001** |
| Married | 455 (60%) | 176 (46%) |  |
| Others | 299 (40%) | 208 (54%) |  |
| **Residence** |  |  | **0.057** |
| City/Town | 292 (39%) | 172 (45%) |  |
| Rural | 462 (61%) | 212 (55%) |  |
| Current smoking, yes | 192 (25%) | 98 (26%) | >0.9 |
| Current alcohol drinking, yes | 158 (21%) | 70 (18%) | 0.3 |
| **Regular exercise** |  |  | **0.8** |
| No | 472 (63%) | 244 (64%) |  |
| Yes | 282 (37%) | 140 (36%) |  |
| **Sleep duration** |  |  | **0.048** |
| <7 h | 191 (25%) | 77 (20%) |  |
| 7-9 h | 428 (57%) | 219 (57%) |  |
| >9 h | 135 (18%) | 87 (23%) |  |
| **Body mass index category, kg/m²** |  |  | **0.021** |
| <18.5 | 148 (20%) | 93 (24%) |  |
| 18.5-23.9 | 433 (57%) | 221 (58%) |  |
| 24.0-27.9 | 140 (19%) | 56 (15%) |  |
| ≥28 | 32 (4.2%) | 10 (2.6%) |  |
| **ADL limitations (number of impaired activities)** |  |  | **<0.001** |
| 0 | 729 (97%) | 338 (88%) |  |
| 1 | 13 (1.7%) | 19 (4.9%) |  |
| ≥2 | 12 (1.6%) | 27 (7.0%) |  |
| **Denture use** |  |  | **0.4** |
| No | 635 (84%) | 332 (86%) |  |
| Yes | 119 (16%) | 52 (14%) |  |
| **Hypertension** |  |  | **0.4** |
| No | 563 (75%) | 301 (78%) |  |
| Yes | 179 (24%) | 77 (20%) |  |
| **Diabetes** |  |  | **0.3** |
| No | 718 (95%) | 358 (93%) |  |
| Yes | 27 (3.6%) | 17 (4.4%) |  |
| **Heart disease** |  |  | **0.3** |
| No | 678 (90%) | 335 (87%) |  |
| Yes | 69 (9.2%) | 43 (11%) |  |
| **Stroke** |  |  | **>0.9** |
| No | 696 (92%) | 355 (92%) |  |
| Yes | 47 (6.2%) | 24 (6.3%) |  |
| **Baseline natural teeth, n** |  |  | **0.043** |
| 20 | 324 (43%) | 200 (52%) |  |
| 21 | 68 (9.0%) | 32 (8.3%) |  |
| 22 | 106 (14%) | 52 (14%) |  |
| 23 | 81 (11%) | 32 (8.3%) |  |
| 24 | 175 (23%) | 68 (18%) |  |

Values are median (Q1, Q3) or n (%). P values were calculated using the Kruskal–Wallis test for continuous variables and Pearson’s chi-square test for categorical variables. Categories for missing covariate data were included in model fitting but are not displayed in this table.

**Table S2. Baseline characteristics of participants included in and excluded from the primary analysis.**

| **Variable** | **Included in analysis N = 3,919** | **Excluded before analysis N = 3,823** | ***P* value** |
| --- | --- | --- | --- |
| Age, years | 83 (75, 90) | 92 (86, 100) | <0.001 |
| **Sex** |  |  | **0.003** |
| Female | 2,179 (56%) | 2,255 (59%) |  |
| Male | 1,740 (44%) | 1,568 (41%) |  |
| **Education, years** |  |  | **<0.001** |
| 0 | 2,354 (60%) | 2,623 (69%) |  |
| 1-6 | 1,187 (30%) | 905 (24%) |  |
| ≥7 | 375 (9.6%) | 295 (7.7%) |  |
| **Marital status** |  |  | **<0.001** |
| Married | 1,466 (37%) | 675 (18%) |  |
| Others | 2,453 (63%) | 3,148 (82%) |  |
| **Residence** |  |  | **0.038** |
| City/Town | 1,460 (37%) | 1,513 (40%) |  |
| Rural | 2,459 (63%) | 2,310 (60%) |  |
| Current smoking, yes | 714 (18%) | 514 (13%) | <0.001 |
| Current alcohol drinking, yes | 735 (19%) | 558 (15%) | <0.001 |
| **Regular exercise** |  |  | **<0.001** |
| No | 2,676 (68%) | 3,029 (79%) |  |
| Yes | 1,243 (32%) | 793 (21%) |  |
| **Sleep duration** |  |  | **<0.001** |
| <7 h | 1,080 (28%) | 1,037 (27%) |  |
| 7-9 h | 1,906 (49%) | 1,594 (42%) |  |
| >9 h | 924 (24%) | 1,172 (31%) |  |
| **Body mass index category, kg/m²** |  |  | **<0.001** |
| <18.5 | 1,161 (30%) | 1,448 (38%) |  |
| 18.5-23.9 | 2,177 (56%) | 1,955 (51%) |  |
| 24.0-27.9 | 457 (12%) | 257 (6.7%) |  |
| ≥28 | 91 (2.3%) | 63 (1.6%) |  |
| **ADL limitations (number of impaired activities)** |  |  | **<0.001** |
| 0 | 3,580 (91%) | 2,740 (72%) |  |
| 1 | 174 (4.4%) | 343 (9.0%) |  |
| ≥2 | 165 (4.2%) | 740 (19%) |  |
| **Denture use** |  |  | **<0.001** |
| No | 2,981 (76%) | 3,298 (86%) |  |
| Yes | 938 (24%) | 525 (14%) |  |
| **Hypertension** |  |  | **<0.001** |
| No | 3,009 (77%) | 3,033 (79%) |  |
| Yes | 837 (21%) | 687 (18%) |  |
| **Diabetes** |  |  | **0.003** |
| No | 3,759 (96%) | 3,640 (95%) |  |
| Yes | 100 (2.6%) | 84 (2.2%) |  |
| **Heart disease** |  |  | **0.050** |
| No | 3,508 (90%) | 3,421 (89%) |  |
| Yes | 352 (9.0%) | 318 (8.3%) |  |
| **Stroke** |  |  | **<0.001** |
| No | 3,674 (94%) | 3,503 (92%) |  |
| Yes | 193 (4.9%) | 230 (6.0%) |  |

Values are median (Q1, Q3) or n (%). P values were calculated using the Kruskal–Wallis test for continuous variables and Pearson’s chi-square test for categorical variables. Categories for missing covariate data were included in model fitting but are not displayed in this table.

**Table S3. Baseline characteristics of participants included in and excluded from the secondary analysis in participants with 25–32 baseline teeth.**

| **Variable** | **Included in analysis N = 1,085** | **Excluded before analysis N = 419** | ***P* value** |
| --- | --- | --- | --- |
| Age, years | 71 (68, 79) | 79 (69, 88) | <0.001 |
| **Sex** |  |  | **0.11** |
| Female | 425 (39%) | 145 (35%) |  |
| Male | 660 (61%) | 274 (65%) |  |
| **Education, years** |  |  | **0.4** |
| 0 | 358 (33%) | 154 (37%) |  |
| 1-6 | 466 (43%) | 179 (43%) |  |
| ≥7 | 260 (24%) | 86 (21%) |  |
| **Marital status** |  |  | **<0.001** |
| Married | 741 (68%) | 228 (54%) |  |
| Others | 344 (32%) | 191 (46%) |  |
| **Residence** |  |  | **<0.001** |
| City/Town | 467 (43%) | 221 (53%) |  |
| Rural | 618 (57%) | 198 (47%) |  |
| Current smoking, yes | 300 (28%) | 92 (22%) | 0.029 |
| Current alcohol drinking, yes | 286 (26%) | 91 (22%) | 0.073 |
| **Regular exercise** |  |  | **0.6** |
| No | 600 (55%) | 238 (57%) |  |
| Yes | 485 (45%) | 181 (43%) |  |
| **Sleep duration** |  |  | **0.001** |
| <7 h | 282 (26%) | 108 (26%) |  |
| 7-9 h | 604 (56%) | 210 (50%) |  |
| >9 h | 199 (18%) | 97 (23%) |  |
| **Body mass index category, kg/m²** |  |  | **0.054** |
| <18.5 | 159 (15%) | 75 (18%) |  |
| 18.5-23.9 | 629 (58%) | 230 (55%) |  |
| 24.0-27.9 | 233 (21%) | 85 (20%) |  |
| ≥28 | 57 (5.3%) | 20 (4.8%) |  |
| **ADL limitations (number of impaired activities)** |  |  | **<0.001** |
| 0 | 1,046 (96%) | 348 (83%) |  |
| 1 | 22 (2.0%) | 29 (6.9%) |  |
| ≥2 | 17 (1.6%) | 41 (9.8%) |  |
| **Denture use** |  |  | **0.8** |
| No | 929 (86%) | 362 (86%) |  |
| Yes | 156 (14%) | 57 (14%) |  |
| **Hypertension** |  |  | **0.4** |
| No | 783 (72%) | 314 (75%) |  |
| Yes | 277 (26%) | 99 (24%) |  |
| **Diabetes** |  |  | **0.7** |
| No | 1,024 (94%) | 393 (94%) |  |
| Yes | 40 (3.7%) | 19 (4.5%) |  |
| **Heart disease** |  |  | **0.3** |
| No | 946 (87%) | 357 (85%) |  |
| Yes | 119 (11%) | 57 (14%) |  |
| **Stroke** |  |  | **0.062** |
| No | 1,002 (92%) | 373 (89%) |  |
| Yes | 66 (6.1%) | 40 (9.5%) |  |
| **Baseline natural teeth, n** |  |  | **0.2** |
| 25 | 131 (12%) | 39 (9.3%) |  |
| 26 | 172 (16%) | 69 (16%) |  |
| 27 | 83 (7.6%) | 31 (7.4%) |  |
| 28 | 241 (22%) | 112 (27%) |  |
| 29 | 57 (5.3%) | 15 (3.6%) |  |
| 30 | 183 (17%) | 77 (18%) |  |
| 31 | 37 (3.4%) | 18 (4.3%) |  |
| 32 | 181 (17%) | 58 (14%) |  |

Values are median (Q1, Q3) or n (%). P values were calculated using the Kruskal–Wallis test for continuous variables and Pearson’s chi-square test for categorical variables. Categories for missing covariate data were included in model fitting but are not displayed in this table.

**Table S4. Baseline characteristics of participants with 20–24 baseline teeth, by baseline vegetable consumption frequency.**

| **Variable** | **Overall N = 754** | **Lower vegetable consumption frequency N = 75** | **Higher vegetable consumption frequency N = 679** | ***P* value** |
| --- | --- | --- | --- | --- |
| Age, years | 73 (69, 81) | 74 (69, 80) | 73 (69, 81) | >0.9 |
| **Sex** |  |  |  | **0.8** |
| Female | 365 (48%) | 38 (51%) | 327 (48%) |  |
| Male | 389 (52%) | 37 (49%) | 352 (52%) |  |
| **Education, years** |  |  |  | **0.2** |
| 0 | 324 (43%) | 40 (53%) | 284 (42%) |  |
| 1-6 | 306 (41%) | 25 (33%) | 281 (41%) |  |
| ≥7 | 124 (16%) | 10 (13%) | 114 (17%) |  |
| **Marital status** |  |  |  | **>0.9** |
| Married | 455 (60%) | 46 (61%) | 409 (60%) |  |
| Others | 299 (40%) | 29 (39%) | 270 (40%) |  |
| **Residence** |  |  |  | **0.4** |
| City/Town | 292 (39%) | 33 (44%) | 259 (38%) |  |
| Rural | 462 (61%) | 42 (56%) | 420 (62%) |  |
| Current smoking, yes | 192 (25%) | 15 (20%) | 177 (26%) | 0.3 |
| Current alcohol drinking, yes | 158 (21%) | 11 (15%) | 147 (22%) | 0.2 |
| **Regular exercise** |  |  |  | **0.7** |
| No | 472 (63%) | 49 (65%) | 423 (62%) |  |
| Yes | 282 (37%) | 26 (35%) | 256 (38%) |  |
| **Sleep duration** |  |  |  | **0.003** |
| <7 h | 191 (25%) | 9 (12%) | 182 (27%) |  |
| 7-9 h | 428 (57%) | 56 (75%) | 372 (55%) |  |
| >9 h | 135 (18%) | 10 (13%) | 125 (18%) |  |
| **Body mass index category, kg/m²** |  |  |  | **0.8** |
| <18.5 | 148 (20%) | 12 (16%) | 136 (20%) |  |
| 18.5-23.9 | 433 (57%) | 43 (57%) | 390 (57%) |  |
| 24.0-27.9 | 140 (19%) | 17 (23%) | 123 (18%) |  |
| ≥28 | 32 (4.2%) | 3 (4.0%) | 29 (4.3%) |  |
| **ADL limitations (number of impaired activities)** |  |  |  | **0.15** |
| 0 | 729 (97%) | 72 (96%) | 657 (97%) |  |
| 1 | 13 (1.7%) | 3 (4.0%) | 10 (1.5%) |  |
| ≥2 | 12 (1.6%) | 0 (0%) | 12 (1.8%) |  |
| **Denture use** |  |  |  | **0.034** |
| No | 635 (84%) | 70 (93%) | 565 (83%) |  |
| Yes | 119 (16%) | 5 (6.7%) | 114 (17%) |  |
| **Hypertension** |  |  |  | **0.2** |
| No | 563 (75%) | 62 (83%) | 501 (74%) |  |
| Yes | 179 (24%) | 12 (16%) | 167 (25%) |  |
| **Diabetes** |  |  |  | **0.2** |
| No | 718 (95%) | 74 (99%) | 644 (95%) |  |
| Yes | 27 (3.6%) | 0 (0%) | 27 (4.0%) |  |
| **Heart disease** |  |  |  | **0.7** |
| No | 678 (90%) | 69 (92%) | 609 (90%) |  |
| Yes | 69 (9.2%) | 5 (6.7%) | 64 (9.4%) |  |
| **Stroke** |  |  |  | **0.5** |
| No | 696 (92%) | 70 (93%) | 626 (92%) |  |
| Yes | 47 (6.2%) | 3 (4.0%) | 44 (6.5%) |  |
| **Baseline natural teeth, n** |  |  |  | **0.005** |
| 20 | 324 (43%) | 38 (51%) | 286 (42%) |  |
| 21 | 68 (9.0%) | 3 (4.0%) | 65 (9.6%) |  |
| 22 | 106 (14%) | 18 (24%) | 88 (13%) |  |
| 23 | 81 (11%) | 8 (11%) | 73 (11%) |  |
| 24 | 175 (23%) | 8 (11%) | 167 (25%) |  |

Values are median (Q1, Q3) or n (%). P values were calculated using the Kruskal–Wallis test for continuous variables and Pearson’s chi-square test for categorical variables. Categories for missing covariate data were included in model fitting but are not displayed in this table.

**Table S5. Baseline characteristics of participants with 25–32 baseline teeth, by baseline vegetable consumption frequency.**

| **Variable** | **Overall N = 1,085** | **Lower vegetable consumption frequency N = 64** | **Higher vegetable consumption frequency N = 1,021** | ***P* value** |
| --- | --- | --- | --- | --- |
| Age, years | 71 (68, 79) | 74 (69, 86) | 71 (68, 79) | 0.014 |
| **Sex** |  |  |  | **0.14** |
| Female | 425 (39%) | 19 (30%) | 406 (40%) |  |
| Male | 660 (61%) | 45 (70%) | 615 (60%) |  |
| **Education, years** |  |  |  | **0.022** |
| 0 | 358 (33%) | 32 (50%) | 326 (32%) |  |
| 1-6 | 466 (43%) | 23 (36%) | 443 (43%) |  |
| ≥7 | 260 (24%) | 9 (14%) | 251 (25%) |  |
| **Marital status** |  |  |  | **0.023** |
| Married | 741 (68%) | 35 (55%) | 706 (69%) |  |
| Others | 344 (32%) | 29 (45%) | 315 (31%) |  |
| **Residence** |  |  |  | **0.4** |
| City/Town | 467 (43%) | 24 (38%) | 443 (43%) |  |
| Rural | 618 (57%) | 40 (63%) | 578 (57%) |  |
| Current smoking, yes | 300 (28%) | 20 (31%) | 280 (27%) | 0.6 |
| Current alcohol drinking, yes | 286 (26%) | 21 (33%) | 265 (26%) | 0.3 |
| **Regular exercise** |  |  |  | **0.009** |
| No | 600 (55%) | 46 (72%) | 554 (54%) |  |
| Yes | 485 (45%) | 18 (28%) | 467 (46%) |  |
| **Sleep duration** |  |  |  | **0.029** |
| <7 h | 282 (26%) | 18 (28%) | 264 (26%) |  |
| 7-9 h | 604 (56%) | 27 (42%) | 577 (57%) |  |
| >9 h | 199 (18%) | 19 (30%) | 180 (18%) |  |
| **Body mass index category, kg/m²** |  |  |  | **0.2** |
| <18.5 | 159 (15%) | 13 (20%) | 146 (14%) |  |
| 18.5-23.9 | 629 (58%) | 39 (61%) | 590 (58%) |  |
| 24.0-27.9 | 233 (21%) | 12 (19%) | 221 (22%) |  |
| ≥28 | 57 (5.3%) | 0 (0%) | 57 (5.6%) |  |
| **ADL limitations (number of impaired activities)** |  |  |  | **>0.9** |
| 0 | 1,046 (96%) | 62 (97%) | 984 (96%) |  |
| 1 | 22 (2.0%) | 1 (1.6%) | 21 (2.1%) |  |
| ≥2 | 17 (1.6%) | 1 (1.6%) | 16 (1.6%) |  |
| **Denture use** |  |  |  | **0.3** |
| No | 929 (86%) | 58 (91%) | 871 (85%) |  |
| Yes | 156 (14%) | 6 (9.4%) | 150 (15%) |  |
| **Hypertension** |  |  |  | **0.4** |
| No | 783 (72%) | 50 (78%) | 733 (72%) |  |
| Yes | 277 (26%) | 12 (19%) | 265 (26%) |  |
| **Diabetes** |  |  |  | **0.2** |
| No | 1,024 (94%) | 60 (94%) | 964 (94%) |  |
| Yes | 40 (3.7%) | 1 (1.6%) | 39 (3.8%) |  |
| **Heart disease** |  |  |  | **0.2** |
| No | 946 (87%) | 59 (92%) | 887 (87%) |  |
| Yes | 119 (11%) | 3 (4.7%) | 116 (11%) |  |
| **Stroke** |  |  |  | **0.4** |
| No | 1,002 (92%) | 60 (94%) | 942 (92%) |  |
| Yes | 66 (6.1%) | 2 (3.1%) | 64 (6.3%) |  |
| **Baseline natural teeth, n** |  |  |  | **0.2** |
| 25 | 131 (12%) | 12 (19%) | 119 (12%) |  |
| 26 | 172 (16%) | 7 (11%) | 165 (16%) |  |
| 27 | 83 (7.6%) | 6 (9.4%) | 77 (7.5%) |  |
| 28 | 241 (22%) | 19 (30%) | 222 (22%) |  |
| 29 | 57 (5.3%) | 5 (7.8%) | 52 (5.1%) |  |
| 30 | 183 (17%) | 8 (13%) | 175 (17%) |  |
| 31 | 37 (3.4%) | 1 (1.6%) | 36 (3.5%) |  |
| 32 | 181 (17%) | 6 (9.4%) | 175 (17%) |  |

Values are median (Q1, Q3) or n (%). P values were calculated using the Kruskal–Wallis test for continuous variables and Pearson’s chi-square test for categorical variables. Categories for missing covariate data were included in model fitting but are not displayed in this table.

**Table S6. Baseline joint fruit and vegetable consumption frequency categories across the three analysis cohorts.**

| **Baseline joint fruit and vegetable category** | **20–24 baseline teeth** | **1–19 baseline teeth** | **25–32 baseline teeth** |
| --- | --- | --- | --- |
| Lower fruit + Lower vegetables | 68 (9.0%) | 340 (8.7%) | 52 (4.8%) |
| Higher fruit + Lower vegetables | 7 (0.9%) | 33 (0.8%) | 12 (1.1%) |
| Lower fruit + Higher vegetables | 367 (48.7%) | 2,108 (53.8%) | 527 (48.6%) |
| Higher fruit + Higher vegetables | 312 (41.4%) | 1,438 (36.7%) | 494 (45.5%) |

Values are n (%). Percentages were calculated within each analytic cohort.

**Table S7. Sensitivity to alternative parameterization of baseline tooth count.**

| **Cohort** | **Exposure** | **Primary model: linear teeth** | **Primary model: factor teeth** |
| --- | --- | --- | --- |
| 20–24 baseline teeth | Higher vs lower fruit consumption frequency (adjusted for vegetable consumption frequency) | 1.05 (0.87, 1.27); P=0.584 | 1.06 (0.88, 1.27); P=0.554 |
|  | Higher vs lower vegetable consumption frequency (adjusted for fruit consumption frequency) | 0.79 (0.59, 1.05); P=0.108 | 0.80 (0.59, 1.08); P=0.144 |
| 1–19 baseline teeth | Higher vs lower fruit consumption frequency (adjusted for vegetable consumption frequency) | 0.97 (0.86, 1.09); P=0.581 | 0.98 (0.87, 1.10); P=0.698 |
|  | Higher vs lower vegetable consumption frequency (adjusted for fruit consumption frequency) | 0.83 (0.69, 1.00); P=0.044 | 0.83 (0.69, 0.99); P=0.043 |
| 25–32 baseline teeth | Higher vs lower fruit consumption frequency (adjusted for vegetable consumption frequency) | 1.03 (0.86, 1.22); P=0.758 | 1.02 (0.86, 1.21); P=0.840 |
|  | Higher vs lower vegetable consumption frequency (adjusted for fruit consumption frequency) | 1.24 (0.87, 1.77); P=0.240 | 1.28 (0.89, 1.85); P=0.182 |

HR, hazard ratio; CI, confidence interval. Estimates were obtained using the primary adjustment set with mutual adjustment for fruit and vegetable consumption frequency.

**Table S8. Single-exposure models of baseline fruit and vegetable consumption frequency.**

| **Cohort** | **Exposure** | **Primary model** |
| --- | --- | --- |
| 20–24 baseline teeth | Higher vs lower fruit consumption frequency | 1.02 (0.85, 1.23); P=0.809 |
|  | Higher vs lower vegetable consumption frequency | 0.80 (0.60, 1.06); P=0.125 |
| 1–19 baseline teeth | Higher vs lower fruit consumption frequency | 0.95 (0.84, 1.06); P=0.358 |
|  | Higher vs lower vegetable consumption frequency | 0.82 (0.69, 0.98); P=0.032 |
| 25–32 baseline teeth | Higher vs lower fruit consumption frequency | 1.04 (0.87, 1.23); P=0.666 |
|  | Higher vs lower vegetable consumption frequency | 1.24 (0.87, 1.77); P=0.226 |

HR, hazard ratio; CI, confidence interval. Fruit consumption frequency and vegetable consumption frequency were modelled separately using the primary adjustment set.

**Table S9. Sensitivity to alternative definitions of fruit and vegetable consumption frequency.**

| **Cohort** | **Exposure** | **Baseline exposure** | **Time-updated exposure** | **Cumulative-average exposure** |
| --- | --- | --- | --- | --- |
| 20–24 baseline teeth | Higher vs lower fruit consumption frequency (adjusted for vegetable consumption frequency) | 1.05 (0.87, 1.27); P=0.584 | 0.98 (0.81, 1.18); P=0.819 | 1.05 (0.87, 1.27); P=0.605 |
|  | Higher vs lower vegetable consumption frequency (adjusted for fruit consumption frequency) | 0.79 (0.59, 1.05); P=0.108 | 0.82 (0.60, 1.11); P=0.191 | 0.80 (0.57, 1.12); P=0.193 |
| 1–19 baseline teeth | Higher vs lower fruit consumption frequency (adjusted for vegetable consumption frequency) | 0.97 (0.86, 1.09); P=0.581 | 0.98 (0.87, 1.10); P=0.725 | 0.96 (0.85, 1.08); P=0.505 |
|  | Higher vs lower vegetable consumption frequency (adjusted for fruit consumption frequency) | 0.83 (0.69, 1.00); P=0.044 | 0.77 (0.65, 0.91); P=0.002 | 0.78 (0.64, 0.94); P=0.011 |
| 25–32 baseline teeth | Higher vs lower fruit consumption frequency (adjusted for vegetable consumption frequency) | 1.03 (0.86, 1.22); P=0.758 | 0.93 (0.78, 1.11); P=0.412 | 1.01 (0.85, 1.20); P=0.909 |
|  | Higher vs lower vegetable consumption frequency (adjusted for fruit consumption frequency) | 1.24 (0.87, 1.77); P=0.240 | 1.12 (0.79, 1.58); P=0.522 | 1.14 (0.78, 1.65); P=0.506 |

HR, hazard ratio; CI, confidence interval. Estimates were obtained using the primary adjustment set with mutual adjustment for fruit and vegetable consumption frequency.

**Table S10. Additional adjustment for Parkinson’s disease and epilepsy.**

| **Cohort** | **Exposure** | **Extended model plus Parkinson’s disease and epilepsy** |
| --- | --- | --- |
| 20–24 baseline teeth | Higher vs lower fruit consumption frequency (adjusted for vegetable consumption frequency) | 1.06 (0.88, 1.28); P=0.538 |
|  | Higher vs lower vegetable consumption frequency (adjusted for fruit consumption frequency) | 0.80 (0.59, 1.07); P=0.136 |
| 1–19 baseline teeth | Higher vs lower fruit consumption frequency (adjusted for vegetable consumption frequency) | 0.97 (0.86, 1.09); P=0.612 |
|  | Higher vs lower vegetable consumption frequency (adjusted for fruit consumption frequency) | 0.82 (0.69, 0.99); P=0.035 |
| 25–32 baseline teeth | Higher vs lower fruit consumption frequency (adjusted for vegetable consumption frequency) | 1.03 (0.87, 1.23); P=0.721 |
|  | Higher vs lower vegetable consumption frequency (adjusted for fruit consumption frequency) | 1.24 (0.87, 1.76); P=0.238 |

HR, hazard ratio; CI, confidence interval. Estimates were obtained from the extended model with additional adjustment for Parkinson’s disease and epilepsy.

**Table S11. Joint models of baseline fruit and vegetable consumption frequency.**

| **Cohort** | **Exposure** | **Primary model** | **Extended model** |
| --- | --- | --- | --- |
| 20–24 baseline teeth | Higher fruit + Lower vegetables | 0.65 (0.34, 1.25); P=0.193 | 0.55 (0.26, 1.17); P=0.120 |
|  | Lower fruit + Higher vegetables | 0.74 (0.53, 1.02); P=0.066 | 0.74 (0.54, 1.03); P=0.072 |
|  | Higher fruit + Higher vegetables | 0.79 (0.57, 1.10); P=0.166 | 0.81 (0.59, 1.12); P=0.208 |
| 1–19 baseline teeth | Higher fruit + Lower vegetables | 0.62 (0.32, 1.19); P=0.150 | 0.61 (0.32, 1.18); P=0.142 |
|  | Lower fruit + Higher vegetables | 0.80 (0.66, 0.96); P=0.018 | 0.79 (0.66, 0.96); P=0.015 |
|  | Higher fruit + Higher vegetables | 0.78 (0.64, 0.96); P=0.017 | 0.78 (0.64, 0.95); P=0.016 |
| 25–32 baseline teeth | Higher fruit + Lower vegetables | 0.70 (0.29, 1.72); P=0.442 | 0.72 (0.30, 1.71); P=0.457 |
|  | Lower fruit + Higher vegetables | 1.15 (0.77, 1.72); P=0.496 | 1.15 (0.77, 1.72); P=0.482 |
|  | Higher fruit + Higher vegetables | 1.20 (0.80, 1.80); P=0.384 | 1.21 (0.80, 1.81); P=0.366 |

HR, hazard ratio; CI, confidence interval. The reference category was lower fruit plus lower vegetables within each cohort. Primary and extended models correspond to those defined in Table 2.

**Table S12. Denture-stratified association of baseline vegetable consumption frequency with incident edentulism in the primary analysis.**

| **Exposure** | **Denture: No** | **Denture: Yes** | **P for interaction** |
| --- | --- | --- | --- |
| Higher vs lower vegetable consumption frequency (adjusted for fruit consumption frequency) | 0.85 (0.69, 1.04); P=0.116 | 0.72 (0.50, 1.05); P=0.085 | 0.537 |

HR, hazard ratio; CI, confidence interval. Estimates compare higher versus lower vegetable consumption frequency with mutual adjustment for baseline fruit consumption frequency and were obtained from the primary model within denture strata. The interaction p value was derived from the corresponding model including a multiplicative interaction term.

**Table S13.** **Death-related sensitivity analyses of baseline fruit and vegetable consumption frequency.**

| **Cohort** | **Exposure** | **Composite endpoint** | **Death-only endpoint** |
| --- | --- | --- | --- |
| 20–24 baseline teeth | Higher vs lower fruit consumption frequency (adjusted for vegetable consumption frequency) | 1.04 (0.88, 1.24); P=0.624 | 0.91 (0.68, 1.22); P=0.546 |
|  | Higher vs lower vegetable consumption frequency (adjusted for fruit consumption frequency) | 0.82 (0.62, 1.09); P=0.175 | 1.14 (0.75, 1.72); P=0.542 |
| 1–19 baseline teeth | Higher vs lower fruit consumption frequency (adjusted for vegetable consumption frequency) | 0.96 (0.88, 1.04); P=0.307 | 0.90 (0.81, 1.00); P=0.046 |
|  | Higher vs lower vegetable consumption frequency (adjusted for fruit consumption frequency) | 0.94 (0.81, 1.08); P=0.384 | 1.04 (0.88, 1.23); P=0.643 |
| 25–32 baseline teeth | Higher vs lower fruit consumption frequency (adjusted for vegetable consumption frequency) | 1.02 (0.87, 1.19); P=0.818 | 1.00 (0.78, 1.29); P=0.992 |
|  | Higher vs lower vegetable consumption frequency (adjusted for fruit consumption frequency) | 1.21 (0.89, 1.64); P=0.219 | 0.95 (0.60, 1.49); P=0.818 |

HR, hazard ratio; CI, confidence interval. Estimates were obtained from the primary adjustment model. Composite-endpoint analyses treated either the tooth-loss event or death as the event of interest; death-only analyses treated death as the endpoint.

**Table S14. Diagnostics for inclusion weighting in the primary analysis.**

| **Metric** | **Value** |
| --- | --- |
| Baseline-eligible participants, n | 7,740 |
| Included in primary analytic cohort, n | 3,919 |
| Excluded before entry into primary analytic cohort, n | 3,821 |
| Observed inclusion probability | 0.506 |
| Predicted inclusion probability among included, median (Q1, Q3) | 0.614 (0.463, 0.756) |
| Predicted inclusion probability among excluded, median (Q1, Q3) | 0.403 (0.260, 0.547) |
| Stabilized inclusion weight among included, mean (SD) | 0.998 (0.602) |
| Stabilized inclusion weight among included, min | 0.511 |
| Stabilized inclusion weight among included, P1 | 0.562 |
| Stabilized inclusion weight among included, median | 0.825 |
| Stabilized inclusion weight among included, P99 | 3.795 |
| Stabilized inclusion weight among included, max | 7.822 |
| Truncated stabilized inclusion weight, mean (SD) | 0.987 (0.525) |
| Truncated stabilized inclusion weight, min | 0.562 |
| Truncated stabilized inclusion weight, P1 | 0.562 |
| Truncated stabilized inclusion weight, median | 0.825 |
| Truncated stabilized inclusion weight, P99 | 3.794 |
| Truncated stabilized inclusion weight, max | 3.795 |

Included participants were those who entered the primary analytic cohort on the basis of non-missing baseline fruit and vegetable consumption frequency data and at least one follow-up natural teeth count, before application of the sequential follow-up rule used to construct the person-period regression sample. Stabilized inclusion weights were truncated at the 1st and 99th percentiles for the truncated-weight analysis.

**Table S15. Unweighted and inclusion-weighted estimates in the primary analysis.**

| **Exposure** | **Unweighted primary model** | **Inclusion-weighted primary model** | **Truncated inclusion-weighted primary model** |
| --- | --- | --- | --- |
| Higher vs lower fruit consumption frequency (adjusted for vegetable consumption frequency) | 0.97 (0.86, 1.09); P=0.581 | 0.93 (0.81, 1.07); P=0.306 | 0.93 (0.81, 1.07); P=0.300 |
| Higher vs lower vegetable consumption frequency (adjusted for fruit consumption frequency) | 0.83 (0.69, 1.00); P=0.044 | 0.88 (0.71, 1.08); P=0.206 | 0.85 (0.70, 1.04); P=0.113 |

HR, hazard ratio; CI, confidence interval. The inclusion-weighted analyses repeated the primary model using stabilized weights derived from the probability of entering the primary analytic cohort.

**Table S16. Crude cumulative incidence of incident edentulism in the primary analysis, by baseline vegetable consumption frequency.**

| **Baseline vegetable consumption frequency** | **N** | **Cumulative incidence by 2011** | **Cumulative incidence by 2014** | **Cumulative incidence by 2018** |
| --- | --- | --- | --- | --- |
| Lower vegetable consumption frequency | 372 | 29.0% | 37.9% | 40.9% |
| Higher vegetable consumption frequency | 3,516 | 21.8% | 30.0% | 33.2% |

Cumulative incidence was estimated from the unweighted person-period sample for the primary analysis.

**Table S17. Missingness of baseline covariates in the primary cohort.**

| **Covariate** | **Missing data, n** | **Missing data, %** |
| --- | --- | --- |
| Parkinson's disease | 117 | 2.985 |
| Hypertension | 73 | 1.863 |
| Diabetes | 60 | 1.531 |
| Heart disease | 59 | 1.505 |
| Stroke | 52 | 1.327 |
| Body mass index category | 33 | 0.842 |
| Epilepsy | 30 | 0.766 |
| Sleep duration | 9 | 0.230 |
| Education | 3 | 0.077 |
| Activities of daily living limitations | 0 | 0.000 |
| Age | 0 | 0.000 |
| Denture use | 0 | 0.000 |
| Current alcohol drinking | 0 | 0.000 |
| Regular exercise | 0 | 0.000 |
| Marital status | 0 | 0.000 |
| Residence | 0 | 0.000 |
| Sex | 0 | 0.000 |
| Current smoking | 0 | 0.000 |
| Baseline natural teeth count | 0 | 0.000 |

Percentages were calculated among participants in the primary analytic cohort before application of the sequential follow-up rule (n=3,919). Missingness was assessed in the original covariate variables before explicit missing-category coding was applied for model fitting. Parkinson’s disease and epilepsy were used only in an additional sensitivity model.

**Table S18. Complete-case sensitivity analysis for the extended model in the primary cohort.**

| **Exposure contrast** | **Participants, n** | **Events, n** | **Hazard ratio (95% confidence interval); P value** |
| --- | --- | --- | --- |
| **Extended model with explicit missing categories** |  |  |  |
| Higher fruit frequency vs lower fruit frequency (adjusted for vegetable frequency) | 3,888 | 1,319 | 0.97 (0.86, 1.09); P=0.598 |
| Higher vegetable frequency vs lower vegetable frequency (adjusted for fruit frequency) | 3,888 | 1,319 | 0.83 (0.69, 0.99); P=0.040 |
| **Complete-case extended model** |  |  |  |
| Higher fruit frequency vs lower fruit frequency (adjusted for vegetable frequency) | 3,730 | 1,263 | 0.98 (0.86, 1.10); P=0.700 |
| Higher vegetable frequency vs lower vegetable frequency (adjusted for fruit frequency) | 3,730 | 1,263 | 0.82 (0.68, 0.99); P=0.035 |

Estimates were obtained from the extended model with mutual adjustment for fruit and vegetable consumption frequency. The extended model adjusted for age, sex, education, marital status, residence, smoking, alcohol drinking, regular exercise, sleep duration, activities of daily living limitations, body mass index category, baseline natural teeth count, denture use, hypertension, diabetes, heart disease, and stroke. Participants and events refer to the final person-period regression sample after application of the sequential follow-up rule.

**Table S19. Sensitivity analyses using the original ordered fruit and vegetable frequency categories in the primary cohort.**

| **Exposure specification** | **Hazard ratio (95% confidence interval); P value** |
| --- | --- |
| **Ordinal trend model** |  |
| Fruit frequency, per one-category higher level | 0.98 (0.92, 1.05); P=0.613 |
| Vegetable frequency, per one-category higher level | 0.89 (0.83, 0.96); P=0.003 |
| **Four-level factor model** |  |
| Fruit frequency: occasionally vs rarely or never | 1.01 (0.88, 1.17); P=0.857 |
| Fruit frequency: quite often vs rarely or never | 1.00 (0.86, 1.17); P=0.974 |
| Fruit frequency: almost every day vs rarely or never | 0.91 (0.73, 1.15); P=0.437 |
| Vegetable frequency: occasionally vs rarely or never | 1.02 (0.66, 1.59); P=0.927 |
| Vegetable frequency: except during winter vs rarely or never | 0.93 (0.61, 1.41); P=0.728 |
| Vegetable frequency: almost every day vs rarely or never | 0.80 (0.53, 1.21); P=0.300 |

The ordinal trend model assigned monotonic scores according to the original questionnaire order. The four-level factor model used “rarely or never” as the reference category for each dietary exposure. All models mutually adjusted fruit and vegetable frequency and used the extended covariate set. Because the questionnaire responses were ordinal categories rather than continuous intake measures, ordered-category and factor specifications were used rather than spline-based dose–response modelling.

**Table S20. Interval-specific follow-up status in the primary analytic cohort, by baseline vegetable consumption frequency.**

| **Baseline vegetable consumption frequency** | **At risk at interval start, n** | **Incident edentulism, n** | **Death before interval end without observed tooth count, n** | **Missing tooth count or censored, n** | **Observed non-event with continued follow-up, n** |
| --- | --- | --- | --- | --- | --- |
| **2008-2011** |  |  |  |  |  |
| Lower vegetable consumption frequency | 373 | 108 | 0 | 1 | 264 |
| Higher vegetable consumption frequency | 3,546 | 767 | 0 | 30 | 2,749 |
| **2011-2014** |  |  |  |  |  |
| Lower vegetable consumption frequency | 264 | 33 | 92 | 21 | 118 |
| Higher vegetable consumption frequency | 2,749 | 287 | 790 | 219 | 1,453 |
| **2014-2018** |  |  |  |  |  |
| Lower vegetable consumption frequency | 118 | 11 | 33 | 29 | 45 |
| Higher vegetable consumption frequency | 1,453 | 113 | 430 | 354 | 556 |

This table was based on the primary analytic cohort with 1–19 baseline natural teeth, non-missing baseline fruit and vegetable frequency, and at least one observed follow-up tooth count (n=3,919), before application of the sequential follow-up rule used to construct the final person-period regression sample. Participants with missing end-of-interval tooth count were censored at the start of that interval and did not contribute subsequent intervals. Death status is shown when death occurred before the interval end and no end-of-interval tooth count was observed. The zero death count in 2008–2011 reflects the restriction to participants with at least one observed follow-up tooth count and should not be interpreted as absence of mortality in the full baseline-eligible cohort; first-interval deaths without observed tooth count are reported in Table S22.

**Table S21. Interval-stability tests and model-fit summary for the extended model in the primary cohort.**

| **Assessment** | **Degrees of freedom** | **Chi-square** | **P value** | **Value** |
| --- | --- | --- | --- | --- |
| **Interval-stability tests** |  |  |  |  |
| Fruit frequency effect varies across intervals | 2 | 1.283 | 0.527 |  |
| Vegetable frequency effect varies across intervals | 2 | 0.717 | 0.699 |  |
| **Overall model-fit summary** |  |  |  |  |
| Overall observed event proportion |  |  |  | 0.2028 |
| Overall mean predicted probability |  |  |  | 0.2027 |
| Brier score |  |  |  | 0.1514 |

Interval-stability tests were robust Wald tests for fruit-frequency-by-interval and vegetable-frequency-by-interval interaction terms in the extended model. Model-fit summaries were calculated from fitted interval-specific probabilities in the final person-period regression sample. The Brier score is the mean squared difference between the observed interval outcome and the predicted interval probability.

**Table S22. Interval-specific participant status in the full baseline-eligible cohort with 1–19 baseline teeth, by baseline vegetable consumption frequency.**

| **Interval** | **Baseline vegetable consumption frequency** | **At risk at interval start, n** | **Incident edentulism, n** | **Death before interval end without observed tooth count, n** | **Missing tooth count or censored, n** | **Observed non-event with continued follow-up, n** |
| --- | --- | --- | --- | --- | --- | --- |
| **2008-2011** | Low vegetables | 940 | 108 | 380 | 188 | 264 |
|  | High vegetables | 6,800 | 767 | 2,124 | 1,160 | 2,749 |
| **2011-2014** | Low vegetables | 264 | 33 | 92 | 21 | 118 |
|  | High vegetables | 2,749 | 287 | 790 | 219 | 1,453 |
| **2014-2018** | Low vegetables | 118 | 11 | 33 | 29 | 45 |
|  | High vegetables | 1,453 | 113 | 430 | 354 | 556 |

Counts were calculated in the full baseline-eligible cohort with 1–19 baseline teeth and non-missing baseline fruit and vegetable consumption frequency (n=7,740), without requiring an observed follow-up tooth count for cohort entry. Within each interval, participant status was classified as incident edentulism, death before interval end without observed tooth count, missing tooth count or censored, or observed non-event with continued follow-up.

**Supplementary Figure Legends**

**Figure S1. Baseline distributions of fruit and vegetable consumption frequency categories.** Panel A shows fruit consumption frequency and Panel B shows vegetable consumption frequency.


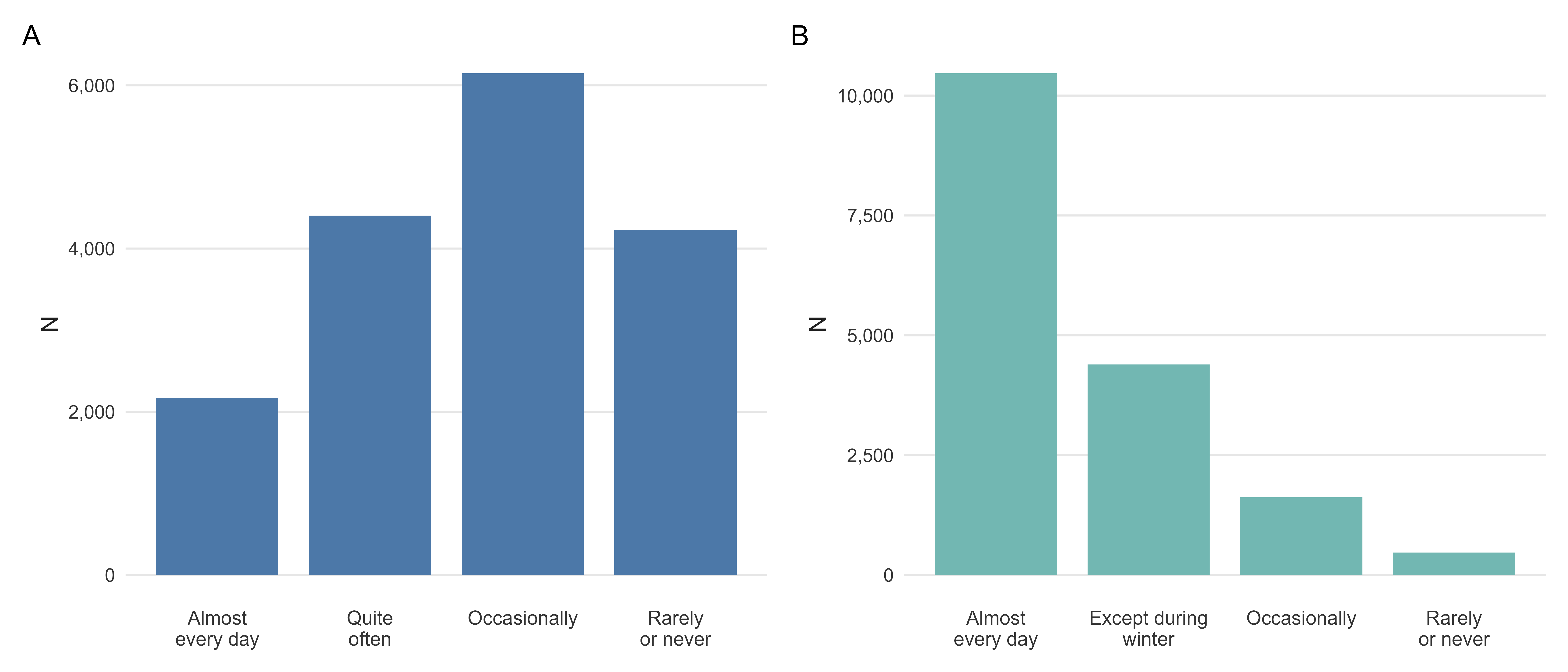


**Figure S2. Crude cumulative incidence by baseline vegetable consumption frequency across the three analysis cohorts.** Estimates were derived from the unweighted person-period samples.


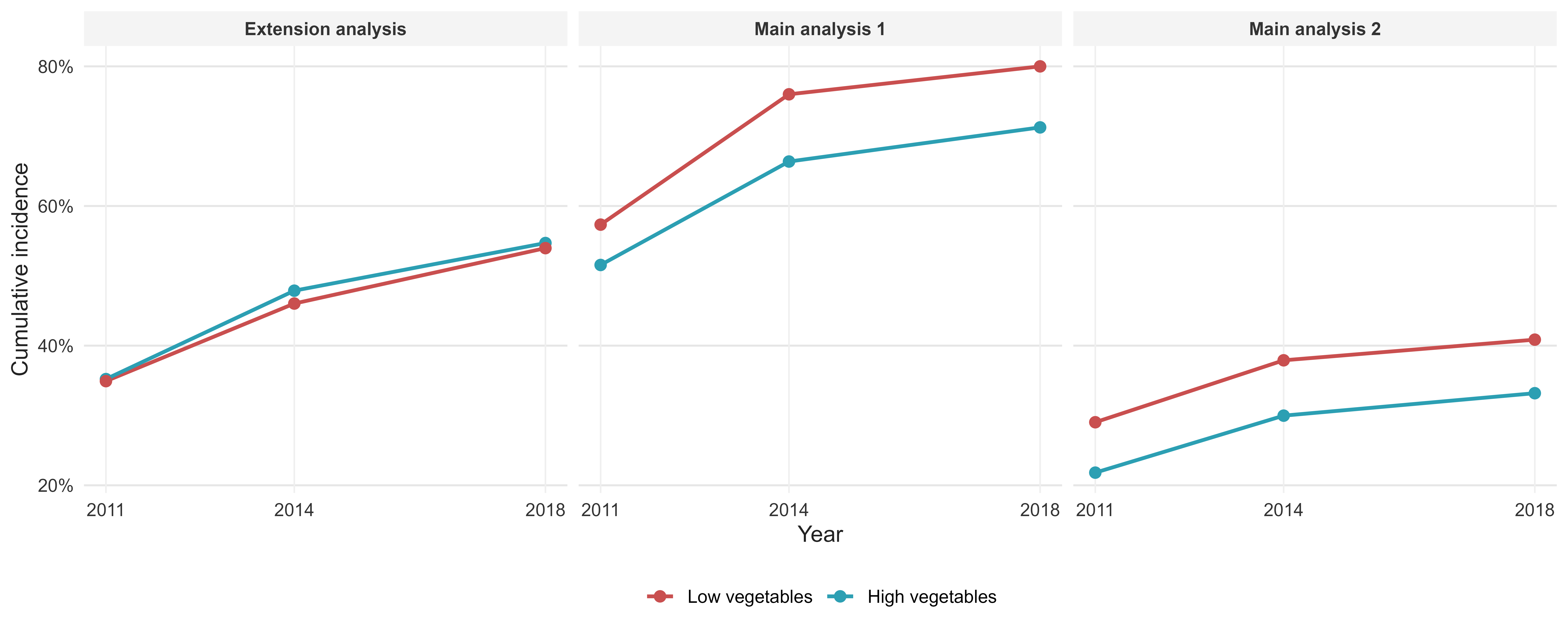


**Figure S3. Joint associations of baseline fruit and vegetable consumption frequency with incident edentulism in the primary analysis.** Points indicate hazard ratios and horizontal bars indicate 95% confidence intervals from the primary adjustment model, with lower fruit plus lower vegetables as the reference category.


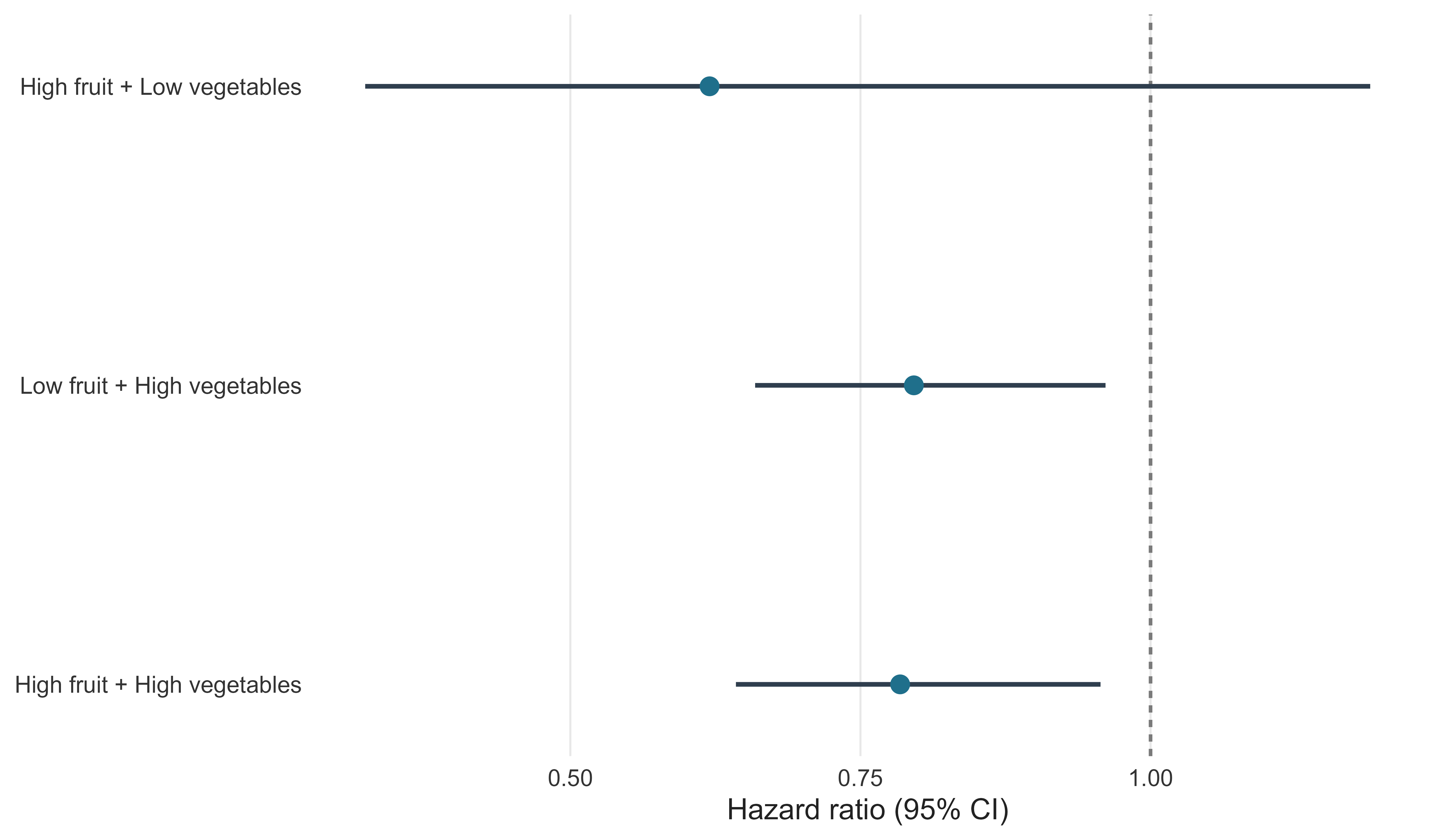

Supplement: Supplementary file 1 [file Table_1.docx]
